# The OsLHY-OsSCL30/OsDHT1 regulatory cascade coordinates heading date and plant height through Pre-mRNA splicing modulation in rice

**DOI:** 10.1016/j.jare.2025.11.023

**Published:** 2025-11-14

**Authors:** Yingying Lu, Yixian Guo, Rui Gao, Jinying Li, Chong Yang, Wenjian Song, Dianxing Wu, Jean Wan Hong Yong, Lujun Yu, Xiaoli Jin

**Affiliations:** a Zhejiang Key Laboratory of Crop Germplasm Innovation and Utilization, College of Agriculture and Biotechnology, Zhejiang University, Hangzhou 310058, China; b State Key Laboratory of Biocontrol, Guangdong Provincial Key Laboratory of Plant Stress Biology, School of Life Sciences, Sun Yat-sen University, Guangzhou 510275, China; c Xianghu Laboratory, Hangzhou 311231, China; d Academy of New Rural Development, Zhejiang University, Hangzhou 310058, China; e State Key Laboratory of Rice Biology, Key Laboratory of the Ministry of Agriculture for Nuclear-Agricultural Sciences, Department of Applied Biosciences, Zhejiang University, Hangzhou 310029, China; f Department of Biosystems and Technology, Swedish University of Agricultural Sciences, 23456 Alnarp, Sweden

**Keywords:** Heading date, Plant height, Splicing, Spliceosome, *OsSCL30*

## Abstract

**Introduction:**

Heading date and plant height are critical agronomic traits in rice, governing climate adaptation and yield. Pre-mRNA splicing is a key post-transcriptional regulatory mechanism influencing plant development and adaptation. While splicing is implicated in regulating these traits, the roles of specific spliceosomal components and their target genes remain elusive.

**Objectives:**

This study aimed to identify and characterize novel splicing factors regulating heading date and plant height in rice, focusing on elucidating the molecular mechanisms involving the spliceosome and its downstream pathways.

**Methods:**

We employed molecular genetics, protein interaction assays (e.g., yeast two-hybrid, bimolecular fluorescence complementation assay), the binding assays of transcript factor to *cis*-element (e.g., yeast one-hybrid, dual-luciferase reporter assay and electrophoretic mobility shift assay), gene expression analysis and splicing efficiency assays (qRT-PCR, RNA immunoprecipitation), and phenotypic and biochemist evaluation of transgenic rice lines (overexpression, gene-editing mutants).

**Results:**

We identified OsSCL30, a rice SR-like splicing factor. Its expression is directly activated by the circadian clock regulator OsLHY, which binds the *OsSCL30* promoter. OsSCL30 interacts with DHT1 to form spliceosomal components. This complex modulates splicing efficiency for specific target genes. *OsSCL30* acts pleiotropically: it delays heading date primarily via the Ghd7–Ehd1–Hd3a/RFT1 pathway and reduces plant height through potential role in strigolactone signaling pathway. Importantly, OsLHY and OsSCL30 form a transcriptional-splicing feedback loop, suggesting a mechanism for maintaining homeostasis.

**Conclusion:**

OsSCL30 functions as a core spliceosomal component, partnering with DHT1, to coordinately regulate rice heading date and plant height. This work uncovers a novel OsLHY-OsSCL30-DHT1 regulatory module integrating transcriptional control and pre-mRNA splicing. Our findings provide a significant theoretical foundation and deliver valuable germplasm resources for enhancing rice adaptation and genetic breeding programs.

## Highlights

• OsSCL30 interacts with DHT1 to form spliceosomal components.
• The spliceosome affects splicing efficiency both directly and indirectly for target genes.
• OsSCL30 delays heading date primarily via the Ghd7–Ehd1–Hd3a/RFT1 pathway.
• OsSCL30 reduces plant height through the strigolactone signaling pathway.
• OsLHY and OsSCL30 form a transcriptional-splicing feedback loop to maintain regulation homeostasis.

## Graphical abstract

**Figure:**
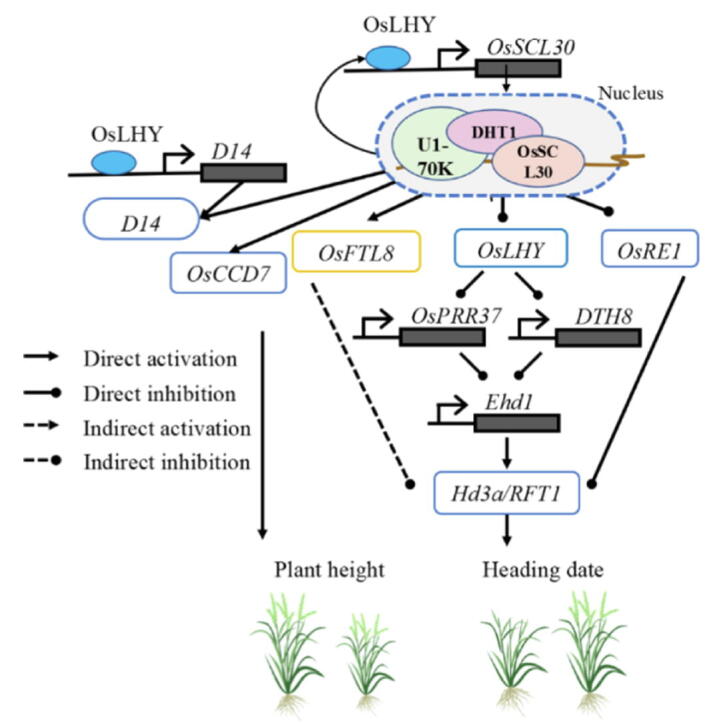

## Introduction

Rice (*Oryza sativa)* is a staple food crop that supports over half of the global population, making its yield and adaptability critical for ensuring global food security, particularly under changing climate conditions [1]. Heading date, or flowering time, plays a central role in rice adaptation to diverse photoperiods and environments, thereby ensuring reproductive success across varied agro-ecological zones [2].

Similar to *Arabidopsis*, florigen (a mobile floral signal) triggers flowering in rice, a critical developmental shift from vegetative to reproductive phase [3,4]. Under appropriate environmental conditions, florigen is synthesized in the leaves and transported to the shoot apical meristem, where it triggers heading [4,5]. The regulation of florigen and heading date in rice is governed by at least two complex genetic networks, including the *OsGI–Hd1–Hd3a* and *Ghd7–Ehd1–Hd3a/RFT1* pathways [6]. In addition to heading date, plant height significantly impacts lodging resistance, biomass allocation, and grain yield, and is regulated by phytohormones such as strigolactones (SLs), brassinosteroids (BRs), and gibberellins (GAs) [[7], [8], [9]]. For example, the semi-dwarf gene *SD1*, encoding a GA20 oxidase (GA20ox), is a key enzyme in the biosynthesis pathway of gibberellin, which affects plant height by regulating ABA synthesis [10]. *OsCCD7* (also known as *HTD1*) encodes a carotenoid cleavage dioxygenase, which is an important enzyme in the biosynthesis of strigolactones. Mutation of the *OsCCD7* gene leads to multi tiller and dwarfism [11,12].

In previous studies, constitutive and alternative splicing have been shown to be involved in flowering and plant height in plants [13,14]. Eukaryotic gene expression requires precise intron excision by the spliceosome, a large ribonucleoprotein complex comprising five snRNPs (U1, U2, U4/U6, U5) and multiple auxiliary proteins [15]. Among these, Serine/Arginine-rich (SR) proteins are key splicing regulators that recognize splicing enhancers (ESEs and ISEs) and recruit core spliceosomal components [16]. SR proteins modulate both constitutive and alternative splicing (AS), and also influence mRNA stability and transcription elongation, thus bridging transcriptional and post-transcriptional regulation [[17], [18], [19], [20], [21], [22]]. The SC35 and SC35-like (SCL) proteins played pleiotropic role in *Arabidopsis*, including leaf phenotype, flowering date, root length via regulation of splicing and transcription of genes [20]. In *Arabidopsis*, the splicing factor SR45 modulates flowering time by regulating the splicing of diverse target genes and participates in ABA signaling and plant defense [23]. SR45, as an APOPTOSIS AND SPLICING ASSOCIATED PROTEIN (ASAP) component was involved in VAL1-mediated FLOWERING LOCUS C (FLC) silencing [21,22].

Similarly, in rice, SR proteins such as SCL28, SCL30, SCL30a, SR32, SR33, RS29, SC25, and RSZ21 interact with DWARF AND HIGH TILLERING1 (DHT1) to modulate the pre-mRNA splicing of *D14*, a gene encoding a strigolactone receptor, leading to increased tillering and decreased plant height, which are typical SL signaling-defective phenotypes [24]. However, the precise molecular mechanisms by which spliceosomal components fine-tune key developmental pathways remain poorly understood. Despite these advances, the functional specialization of SR proteins in crop-specific developmental processes, particularly those regulating heading date and plant height, remains largely uncharacterized.

In our previous study, we identified an SR protein, OsSCL26, as a regulator of heading date by participating in the post-transcriptional splicing of target genes [25]. RNA-seq analysis revealed significant differences of *OsSCL30* expression between the wild-type (WT) and *osscl26* mutant. Like OsSCL26, OsSCL30 is classified as a canonical SR protein according to the previous nomenclature [26]. Based on these findings, we generated both gene-edited and overexpression lines of *OsSCL30*. In this study, we identify *OsSCL30*, the member from SR protein family, mediating heading date and plant height in rice. We demonstrate that: (1) OsLHY binds to the *OsSCL30* promoter directly, activating its transcription and linking circadian rhythms to splicing regulation. (2) *OsSCL30* interacts with DHT1, a spliceosomal component, to form a functional complex that modulates the splicing of key genes—affecting flowering time via the photoperiodic Ghd7–Ehd1–Hd3a/RFT1 pathway and plant height through potential role in strigolactone signaling pathway. (3) A feedback loop exists between OsLHY and *OsSCL30*: OsLHY enhances *OsSCL30* transcription, while *OsSCL30* modulates the splicing of *OsLHY* pre-mRNA, maintaining homeostatic expression levels. Our findings reveal a novel regulatory module in which the OsSCL30–DHT1 spliceosome integrates transcriptional regulation (via OsLHY) with post-transcriptional control of photoperiodic and hormonal (SL) signaling to optimize heading date and plant height. This study advances our understanding of splicing-mediated regulation in crop adaptation and offers valuable genetic resources for high-yield, climate-resilient rice cultivars.

## Materials and methods

### Plant materials and growth conditions

The rice (*Oryza sativa* L.) varieties used in this work included gene-editing lines (*scl30-1*, *scl30-2*, *ftl8*-1 and *ftl8*-2) by CRISPR/Cas9 technology and overexpression lines of OsSCL30 (OE#1, OE#2) were grown in the experimental field of Zhejiang University in Changxing under natural growth conditions. The materials were grown in the chambers for control long-day (CLD) (14 h light/28℃; 10 h dark/24℃) and control short-day (CSD) (10 h light, 28℃/14 h dark, 24℃) conditions. The light intensity in the incubators is 600 μmol·m^−2^·s^−1^, and the humidity is 70 %.

### Vector construction and rice transformation

We created gene-editing lines of *OsSCL30* and *OsFTL8* using CRISPR/Cas technology following Jin’s methods with modification [27]. Firstly, the target sequences for editing were selected using the target-designing website (http://skl.scau.edu.cn/) [28] and these target sequences were inserted into pC1300-Cas9 vector using the SK-gRNA vector. For overexpression studies, the entire coding sequence of *OsSCL30* fused with a 3 × flag tag was inserted into the pUN1301 vector, which was driven by the Ubi promoter. The specific primers OsSCL30-P-F and OsSCL30-P-R were designed to amplify the OsSCL30 promoter from the Ricedata website (https://www.ricedata.cn/). The purified DNA fragment was ligated to the pCAMBIA1381Z vector cleaved by *Bam*HⅠ and *Hin*dⅢ enzymes using T_4_ ligase. After sequencing and alignment, the pOsSCL30::GUS vector was obtained. The *Agrobacterium tumefaciens EHA105* carrying theses vectors were ***introduced into callus.*** The primer pairs used for vector constructions are listed in Supplemental Table S1.

### RNA Isolation and qRT–PCR Analysis

Following the manufacturer's protocol (Invitrogen, Carlsbad, CA, USA), we isolated total RNA with TRIzol reagent. One μg RNA was used to synthesize the first strand cDNA by the PrimeScript RT reagent Kit with gDNA Eraser (Takara, Japan). The cDNA was used for RT-PCR by PrimerSTAR MaxDNA Polymerase (Takara, Shiga, Japan). The qRT-PCR was performed with SYBR Green qPCR Master Mix (TOROIVD, Japan) on LightCycler 480 II (Roche, South San Francisco, CA, USA). The *OsActin* was used as the internal control and the relative expression levels were calculated using the 2^−ΔΔCT^ method and Student's t −test.

### GUS analysis

The different tissues pOsSCL30::GUS transgenic plants were harvested and ***immersed in the GU***S buffer (Cat: SL7160-3–50 ml, Beijing Coolaber company). The samples were incubated at 37 ℃ for 24 h in the dark, then decolorized with 75 % (v/v) ethanol, and finally ***images were captured using a Nikon AZ100 camera.***

### Yeast one-hybrid (Y1H) assay

The *cis*-elements, 3 × AAAAATCT, fragment of the *OsSCL30* promoter was cloned into pAbAi vector (Clontech). The plasmid was then linearized by digestion with *Bst*BI. The linearized plasmid was purified and transformed into the Y1HGold ([genotype, e.g., MATa, ura3-52, his3-200, ade2-101, trp1-901, leu2-3, 112, gal4Δ, gal80Δ, met–, MEL1]) yeast strain. Transformation mixtures were plated on SD medium lacking uracil (SD/-Ura) to select for yeast cells in which the linearized plasmid had been integrated into the genome. The integrity of the bait sequence in the final pAbAi plasmid was confirmed by sequencing. Furthermore, the correct genomic integration of the bait construct was verified by colony PCR and sequencing of the amplified the fragment. Positive colonies were selected and referred to as the bait strain. To establish the minimum concentration of AbA required to completely suppress the background growth of the bait strain in the absence of any interacting protein, the bait strain was grown and serially diluted. The diluted cultures were spotted onto SD/-Ura plates supplemented with increasing concentrations of AbA. After incubation at 30℃ for 3–5 days, the lowest AbA concentration (600 ng/mL) that completely inhibited yeast growth was determined. This optimized concentration was used for all further experiment. The full-length *OsLHY* coding sequence was inserted into pGADT7. The bait strain was transformed with the pGADT7-Rec vector. The transformation mixture was plated onto SD/-Leu medium and containing the pre-determined optimal AbA concentration. The plates were incubated at 30 °C for 3–5 days. The background expression level of AbA in the test bait strain and the point-to-point validation of yeast heterozygosity were both based on Matchmaker's reference® Gold Yeast One −Hybrid Library Screening System User Manual (Clontech).

### Dual-Luciferase Reporter Assay (DLuc)

The promoter of *OsSCL30* was ligated into pGreen0800-LUC vector upstream of firefly luciferase. While the effector vector carried the full-length CDS of *OsLHY* under the control of CaMV35S promoter. All constructs were transferred into GV3101-pSoup Agrobacterium Competent Cells and co-transfected into tobacco leaves. The activity of Luciferase was detected after 24 h post-transfection with Dual-Glo® Luciferase Assay System (Promega) on a GloMax® Navigator Microplate Luminometer. The firefly luciferase activity was normalized to the internal control *Renilla* luciferase activity. The data were captured during the same session using same leaf, identical microscope, camera, and software settings. The data presented were from three independent biological replicates (n = 3) by Student’s T test.

### Protien Expression and Electrophoretic mobility shift assay (EMSA)

The full-length CDS of OsLHY was fused with GST tag in pGEX-4 T-1 vector. Then, the fused protein was expressed in *E. coli* Rosetta (DE3) cells, and purified by GST-tag protein purification kit (Beyotime, China). A 5′-biotinylated oligonucleotide (5′- aaaattgtgaaaaatctacgcttcaacatt-3′) was used as the probes. We also design the competitor probe without 5′-biotinylation and mutant probe 5′-biotin-aaaattgtgccccccccacgcttcaacatt-3′. The purified protein and DNA were mixed in binding buffer (0.2  mg ml^−1^ BSA, 25 % glycerol, 1  mM dithiothreitol (DTT), 10  mM MgCl_2_, 50  ng ml^−1^ deoxyribonucleic acid, 40  mM KCl, and 100  mM Tris–HCl [pH 7.9]). The reaction was performed at 25 °C for 30  min. Then, we run the entire reaction mixture on a 6 % non-denaturing polyacrylamide gel for 1 h at 4 °C at 60 V and transferred to nylon membranes (Pall Corporation). Finally, the signals were visualized with reagents included in the kit and ChemiDoc XRS (Bio-Rad Laboratories, UAS).

### Subcellular localization

The full-length *OsSCL30* coding region, both with and without the signal peptide, were fused with green fluorescent protein (GFP) in the pCAMBIA1305 vector driven by the CaMV35S promoter. Then, the OsSCL30-GFP were transformed into *Nicotiana benthamiana* leaves, and subjected to staining with the DNA-specific fluorochrome, DAPI. The OsSCL30-GFP was excited with a 488 nm laser and detected at 500–540 nm, while DAPI was excited with a 405 nm laser and detected at 430–470 nm. For co-localization, OsSCL30-GFP was co-expressed with NLS-RFP, which serves as a nuclear marker in *N. benthamiana* leaves. After 48 h, the fluorescent signals from transformed leaves were observed under the excitation wavelengths of 488 and 583 nm for GFP and mCherry, respectively. The emitted signals were detected at wavelength window of 500–540 nm and 570–620 nm, respectively. The fluorescence signals of GFP, DAPI and RFP were captured using a confocal laser scanning microscope (FV3000, Olympus, Japan).

### Yeast 2-Hybrid (Y2H) assay

Full-length CDS of *OsSCL30* was ligated to the pGBKT7 vector cleaved by *Nco*Ⅰ and *Eco*RⅠ enzymes using T_4_ ligase, and the bait vector was obtained after sequencing and alignment. The full-length CDS of *DHT1* was inserted into pGADT7 vector. Autoactivation testing was rigorously performed for each bait and prey construct. The pGBKT7-OsSCL30 bait plasmid was co-transformed with the empty pGADT7 vector to test for autoactivation of the reporter system. Likewise, the pGADT7-DHT1 prey plasmid was co-transformed with the empty pGBKT7 vector. No autoactivation was observed for either construct under the selective conditions. For the interaction assay, the bait and prey vectors (pGBKT7-OsSCL30 and pGADT7-DHT1) were co-transformed. The following control combinations were simultaneously analyzed: the pGADT7-AgT + pGBKT7-p53 was used as the positive control, and pGADT7-AgT and pGBKT7-lamC as negative control. The cultures grew on SD − Leu/−Trp plates and selective media agar plates. The dilution was spotted on co-transformation medium agar plates using pipette, and were incubated at 30 °C, and observed within 6 days.

### Bimolecular Fluorescence Complementation (BiFC) Assay

The full-length CDSs of *OsSCL30* and *DTH1* were cloned into pCAMBIA1300-35S-nYFP and pCAMBIA1300-35S-cYFP plasmids, respectively. The *Agrobacterium tumefaciens* strain GV3101 harboring the constructs was co-infiltrated into *Nicotiana benthamiana* leaves. We performed the control experiments including YFP^N^ + DHT1-YFP^C^, OsSCL30-YFP^N^ + YFP^C^ and YFP^N^ + YFP^C^. Fluorescence signals were observed 48–72 h post-infiltration using a confocal laser scanning microscope (FV3000, Olympus, Japan) at a wavelength range of 500 nm to 542 nm. Negative controls included empty vector combinations and single construct infiltrations.

Rice protoplasts were prepared according to previous method described and the constructs were transiently expressed using the PEG-mediated transformation method following the method [29]. After 12–16 h incubation, the signal was observed using a confocal laser scanning microscope (FV3000, Olympus, Japan) at a wavelength range of 500–542 nm.

### Luciferase Complementation Imaging (LCI) Assay

The full-length CDS of *OsSCL30* was cloned into the pCAMBIA1300-nLUC vector to generate an OsSCL30-nLUC fusion construct. Similarly, the full-length CDS of *DHT1* was cloned into the pCAMBIA1300-cLUC vector to obtain a DHT1-cLUC fusion construct. The resulting constructs, along with the corresponding empty vector controls, were introduced into *Agrobacterium tumefaciens* strain GV3101. Agrobacterial cultures carrying the constructs were grown to an OD_600_ of 0.8, harvested, and resuspended in infiltration buffer (10 mM MgCl_2_, 200 μM acetosyringone, 10 mM MES, pH 5.6). Equal volumes of Agrobacterium cultures carrying OsSCL30-nLUC and DHT1-cLUC were co-infiltrated into tobacco (*Nicotiana benthamiana*) leaves using a needleless syringe. Then, we incubated the plants in the dark for 24 h and normal growth condition (25 °C, 16 h light/8h dark) for 48 h. Luciferase activity was detected by spraying 1 mM D-luciferin onto the infiltrated leaf areas, followed by imaging using PlantView100. The data were captured during the same session using same leaf, identical microscope, camera, and software settings. The data presented were from three independent biological replicates (n = 3) by Student’s T test.

### RNA-seq and RNA-seq data analysis

RNA seq analysis was performed on the mixed samples of leaves of wild-type Nipponbare and gene-editing line, *scl30-1*, before heading, and the sequencing was completed by Shanghai Meiji Biotechnology Co., Ltd. Using Illumina sequencing platform. Raw sequencing reads were assessed for quality using fastx_toolkit (Version 0.0.14). High-quality reads were aligned to the reference genome (Weblink: http://rice.plantbiology.msu.edu/analyses_search_locus.shtml) using STAR software (version 2.5.3a). Read counts were used as input for differential expression analysis with DESeq2 software (Version 1.24.0). Genes with an adjusted p-value (FDR) < 0.05 and an absolute log2 fold change (|log2FC|) > 1 were considered statistically significantly differentially expressed. Functional enrichment was performed on the resulting sets of differentially expressed and spliced genes. We used rMATS software (version 3.2.5) to analyze the different AS events. The datasets generated and analyzed during the current study are available in the Sequence Read Archive database repository (Accession No, GSE307287).

### Western blotting

Total proteins were extracted from the Nipponbare and *scl30-1* mutant with extraction buffer (150 mM NaCl, 1 mM EDTA, 50 mM Tris-HCl pH 7.5, protease inhibitor cocktail and 1 % Triton X-100). The supernatant was collected after centrifugation at 12,000 × g for 15 min at 4 °C. Then 30 µg proteins were separated by 10 % SDS-PAGE and put on a polyvinylidene fluoride Membrane. The membrane was blocked with 5 % non-fat milk in TBST and incubated with anti-D14 primary antibody (1:2000) overnight at 4 °C, HRP-conjugated secondary antibody (1:5000) (Cat No. SA00001-2, Proteintech Group, Inc.) for 1 h at room temperature. Before western blotting, a His-fused polypeptide (amino acids 153–302 of D14) was expressed in *BM*Rosetta (DE3) cells and purified. The polyclonal antibodies of D14 were produced by injecting the fused protein into rats. The anti-Tubulin antibody (Cat No. 80762–1-RR Proteintech Group, Inc.) was used as control. Signals were detected and imaged with a chemiluminescence system. Anti-actin antibody was used as a loading control.

### RNA immunoprecipitation assay

RNA immunoprecipitation (RIP) assays were performed as described previously [25].

### Strigolactone analysis

We measured the levels of epi-5DS from rice root reference to the method as described [30]. Briefly, the rice plants were grown in hydroponic culture. Root exudates were collected by transferring seedlings to fresh medium containing 1 ng d_6_-5DS (internal standard) and incubating for 24 h. Exudates were adsorbed onto Oasis HLB 3 cc cartridges, washed with deionized water, and eluted with acetone. Extracts were dried under nitrogen, reconstituted in acetonitrile, and detected by ultra-performance liquid chromatography-tandem mass spectrometry (UPLC-MS/MS).

## Results

### OsSCL30 regulates early-heading and semi-dwarf traits

**Fig. 1:**
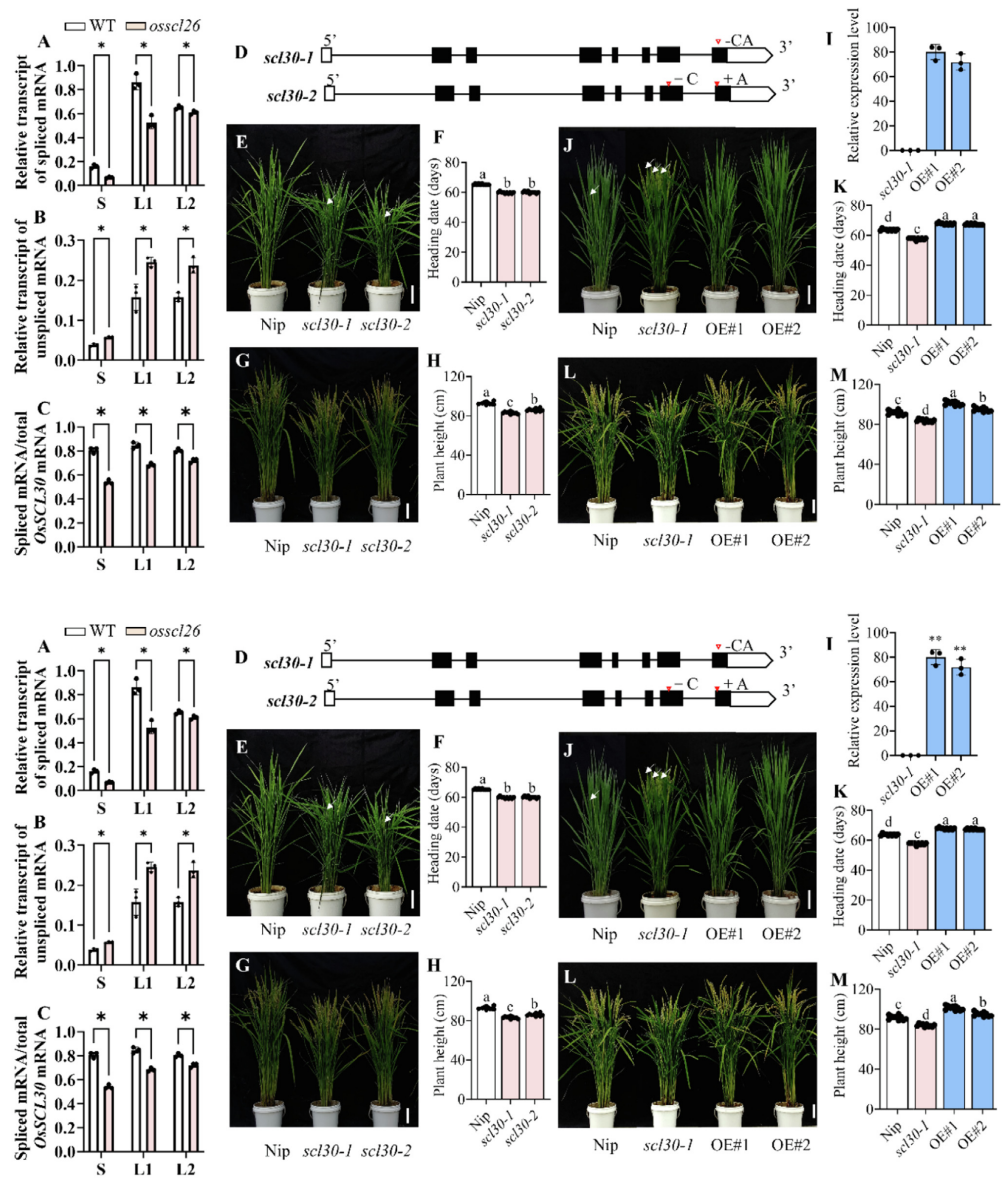
The splicing of *OsSCL30* and phenotypes among Nipponbare, gene-editing and overexpression lines of *OsSCL30*. A-B) Relative transcript of spliced and unspliced mRNAs of *OsSCL30* in the WT and *osscl26* mutant. C) The ratio of spliced mRNA and total *OsSCL30* mRNA. A-C) and I) Significant differences were identified at 5％(*) and 1 % (**) probability levels with three biological replicates by Student’S t-test.D) The mutation sites on two gene-editing lines created by CRISPR/Cas9 system. The unfilled boxes, the filled boxes, lines, and red triangle represent UTR (5′-UTR and 3′-UTR), the CDS, introns, and editing sites, respectively. (+), and (−) represent the insertion sites and the deletion site at two *scl30* mutants, respectively. Comparison of heading date (E-F) and plant height (G-H) between Nip and gene-editing lines in 2022. I) The expression level of *OsSCL30* in two overexpression lines. J-K) Comparison of heading date (J-K) and plant height (L-M) among Nip, gene-editing and overexpression lines in 2023. Nip: Nipponbare; *scl30-1*, *scl30-2*: two gene editing lines of *OsSCL30*. Data in panels F, H, K and M are presented as means ± SD with 10 samples. Different letters above error bars indicate significant differences among the genotypes at *p* ≤ 0.05, as determined by one-way ANOVA analysis. S, L1 and L2 in (A-C) represent stem, leaf at seeding stage and filling stage. Scale bars in (E), (G), (J) and (L) represent 10 cm.

In our previous study, we identified an *osscl26* mutant, which exhibited slender grains, early heading date and good appearance quality compared with its wild type [25]. According to the RNA-seq analysis between the WT and *osscl26* mutant, we found *OsSCL30* underwent splicing and expression difference, which is the member of same SCL subfamily with *OsSCL26*. Compared to the WT, the spliced and unspliced mRNA of *OsSCL30* decreased along with decrease of the splicing effect (Fig. 1, Fig. 1 and Fig. S1A). To further identify the function of *OsSCL30*, we generated the knocked-out lines using CRISPR/Cas9 approach (Fig. 1D and Fig. S1). The gene-editing line *scl30-1* had a deletion of 2 bases (CA) at the 687th, resulting in premature termination, while the *scl30-2* has a deletion of C at the 508th and an A insertion of base at the 688th, resulting in a frameshift mutation. Interestingly, we found that the gene edited lines *scl30-1* and *scl30-2* showed significant differences in heading stage and plant height compare to the WT. The *scl30-1* and *scl30-2* stared to heading 5–6 days earlier than the Nipponbare under natural photoperiod and temperature conditions (Fig. 1, Fig. 1; Fig. S2), while the plant height decreased by 7.36–10.40 %, respectively (Fig. 1, Fig. 1). To further validate the function of *OsSCL30* in regulating heading date and plant height, we over-expressed *OsSCL30* in the *scl30-1* (Fig. 1I). The results presented that there were significant differences in heading date and plant height between overexpression lines and *scl30-1*, which to some extent complement the phenotype of the gene edited line *scl30-1*. The heading date of the overexpression lines were delayed by 9–11 days, and the plant height increased by 13.32 % to 20.74 % (Fig. 1, Fig. 1).

Besides the heading date and plant height, there were significant differences between the gene-edited lines and Nipponbare in traits such as effective tiller number, panicle length, grain weight reduction, yield per plant, grain length, grain width, grain thickness, and ratio of grain length to grain width. The Effective tiller number, grain length, the ratio of grain length to grain width increased. In contrast, the panicle length, grain width, gain thickness, 1000-grains weight and yield per plant were significantly lower (Table S2). The results indicated that loss-of-function of *OsSCL30* could lead to an earlier heading date and altered plant height, but with a slight reduction in yield. The two overexpression lines partially complemented the phenotypic characteristics associated with *scl30-1* (Table S3). Thus, we propose that *OsSCL30* exhibits functional pleiotropic effect.

The gene-edited line *scl30-1* was crossed with the WT (Nipponbare) to generate F_1_. Phenotypic analysis of the F_1_ plants revealed that their heading date was 5–6 days later than that of *scl30-1*, but showed no significant difference compared to Nipponbare. The plant height of F_1_ was 10.54 cm taller than *scl30-1*, but exhibited no significant difference from Nipponbare (Fig. S3) as well. Subsequently, we observed the phenotypic segregation in the F_2_ population, which was derived from F_1_ self-pollination. The results of the genetic analysis showed a segregation of 981 wt and 305 mutant types (*χ*^2^ = 1.13 < *χ*^2^_0.05_ = 3.84), indicating that *OsSCL30* is a dominant gene regulating delayed heading date, and increasing plant height in rice.

### OsSCL30 expression is regulated by OsLHY

**Fig. 2:**
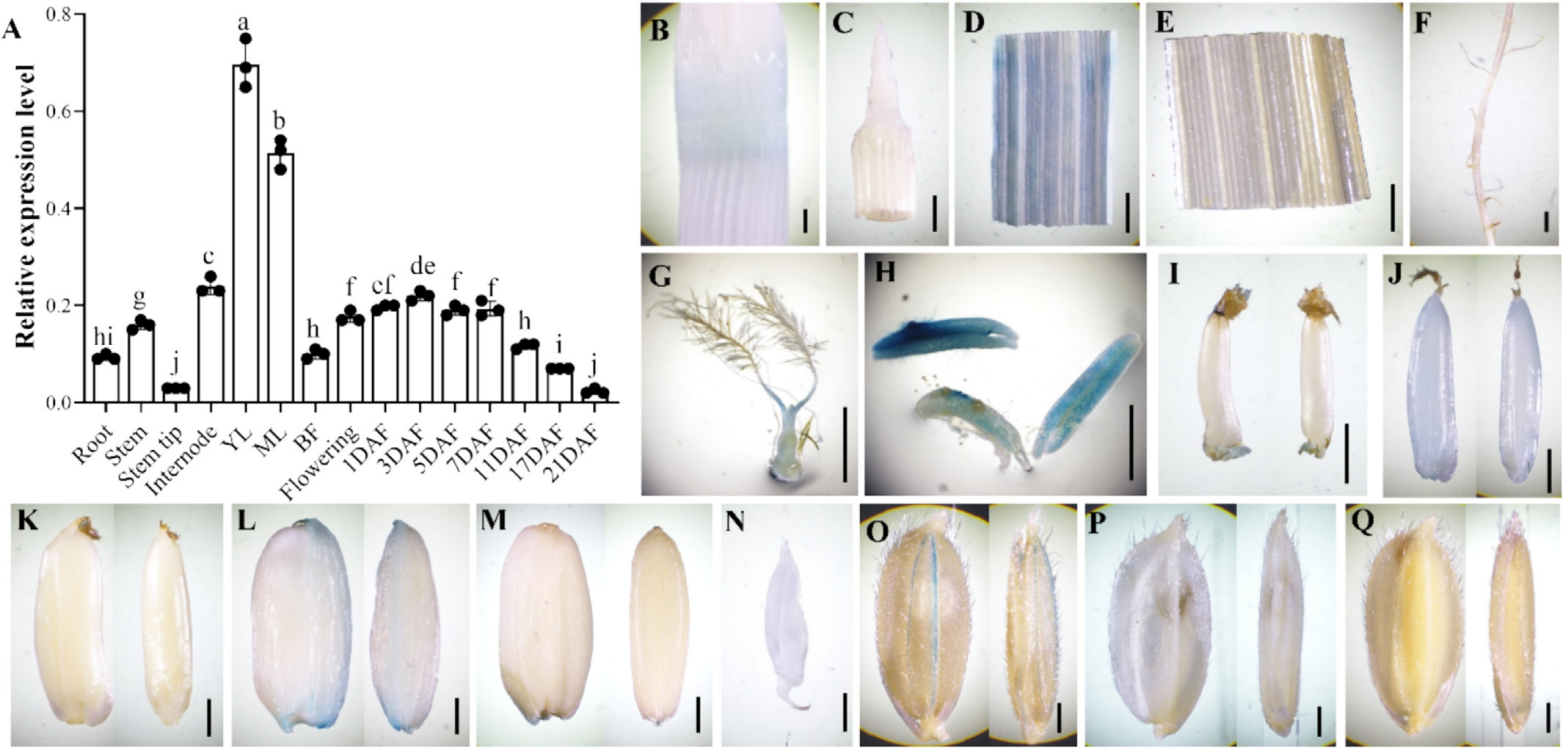
Expression pattern of *OsSCL30*. A) Expression pattern of the *OsSCL30* gene in various tissues of rice plants. The sampling periods include the root, stem, stem tip, internode, young leaves (YL), mature leaves (ML), the spikelets before flowering (BF), at flowering and on the 1st, 3rd, 5th, 7th, 11th, 17th and 21st days after flowering (DAF). Data are presented as means ± SD, and each group of data has three biological repetitions. Different letters above error bars indicate significant differences among the genotypes at *p* ≤ 0.05, as determined by one-way ANOVA analysis. The analysis of *OsSCL30*_promoter_:GUS in the internode (B), stem tip (C), young leaf blade (D), mature leaf blade (E), root (F), pistil (G), stamen (H), the caryopsis at first day (I), 3th day (J), 5th day (K), 7th day (L), 11th day (M) after fertilization, the spikelet before flowering (N), and the spikelet at 1 first day (O), 3th day (P), 5th day (Q) after fertilization. The left and right images represent the front and side of the caryopsis. Scale bars = 1 mm in B-Q.

The expression patterns of *OsSCL30* in various tissues were analyzed using qRT-PCR. Consistent with a previous report [31], our results indicated that *OsSCL30* was expressed in all examined tissues, with relatively higher expression levels in leaves, particularly in young leaves. In contrast, its expression was very low in roots, stems, and spikelets at different stages (Fig. 2A). Statistical analysis revealed significant differences in expression across these tissues (P < 0.05). To further investigate the expression characteristics of the *OsSCL30* gene, we generated transgenic lines harboring the GUS reporter gene driven by *OsSCL30* promoter (Fig. 2, Fig. 2). Histochemical staining revealed strong GUS activity in pre-heading leaves and stamens, while weaker GUS signal was observed in internodes, heading-stage leaves, pistils, and caryopses at 1, 3, 5, 7, and 11 days after fertilization (DAF), as well as in spikelets at 1, 3, and 5 DAF. Almost no GUS signal was detected in shoot apices, roots, or pre-flowering spikelets. Both qRT-PCR and GUS staining further confirmed that *OsSCL30* was highly expressed in young and mature leaves, which is consistent with the published data [31], while the GUS-based spatial and temporal expression patterns represent visualization expression of *OsSCL30* in different tissues.

To analyze the *cis*-elements and transcript factors of the *OsSCL30* promoter, we downloaded the 2677 bp sequence upstream of the ATG of the *OsSCL30* gene from the website of Rice Genome Annotation Project (https://rice.uga.edu/). The websites of PlantPAN (http://plantpan.itps.ncku.edu.tw/) and PlantCARE (https://bioinformatics.psb.ugent.be/webtools/plantcare/html/) were used to predict transcription factors and corresponding *cis*-elements on the *OsSCL30* promoter. We found the *OsSCL30* promoter contained numerous light-responsive elements, including light-responsive DNA modules (Box 4 and ATCT-motif), G-box, TCT-motif, TCCC-motif, and GTGGC-motif, and AE-box. It also includes many hormone-responsive elements, such as *cis*-element related to MeJA (CGTCA-motif and TGACG-motif), gibberellin (P-box), auxin (TGA-element) and abscisic acid (ABRE). Furthermore, the *OsSCL30* promoter contains stress-responsive elements and tissue-specific expression elements (Table S4). Therefore, the *cis*-elements of the *OsSCL30* promoter could modulate the growth, development, and environmental responses of rice plants by participating in the regulation of gene expression.

**Fig. 3:**
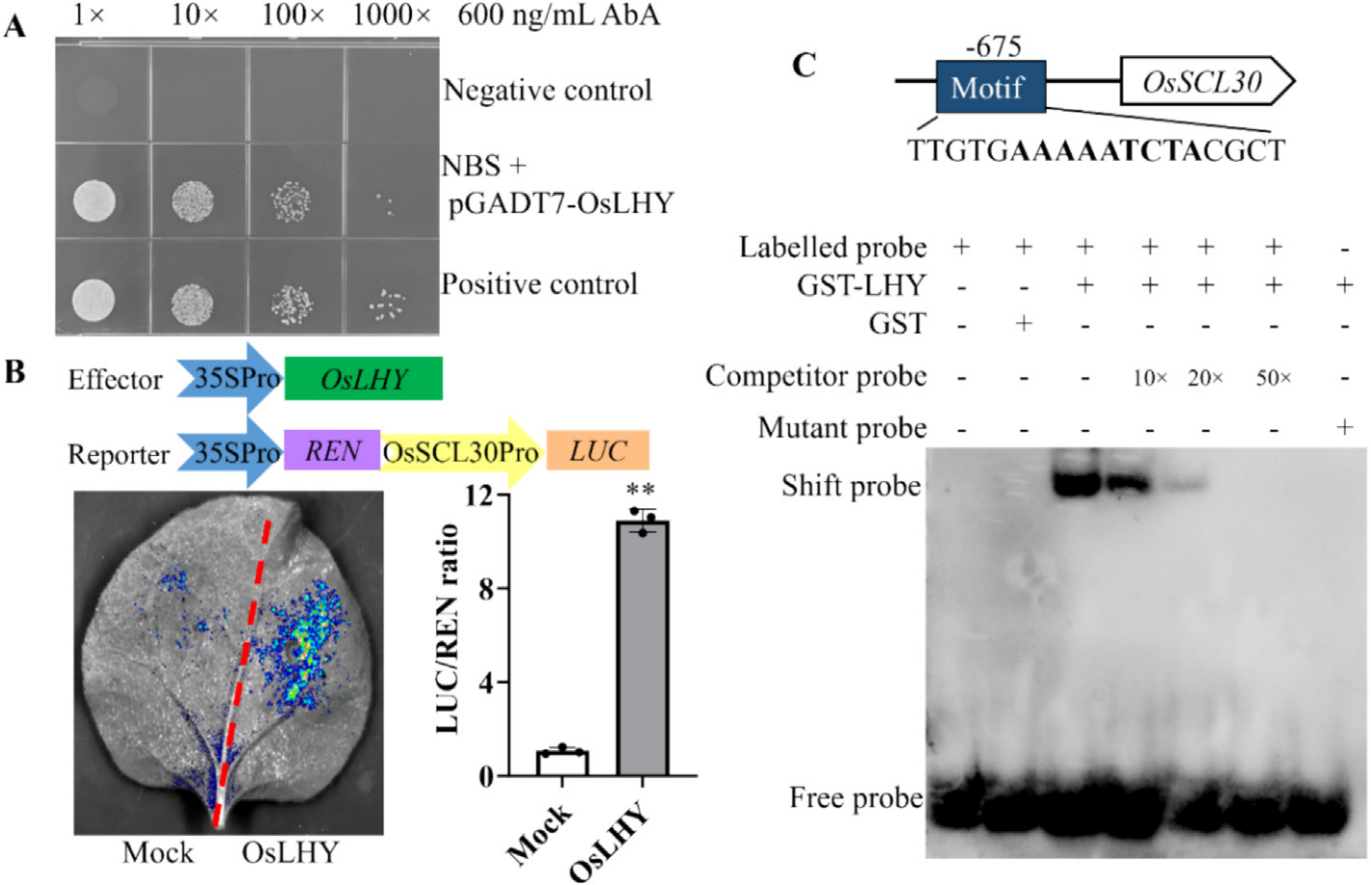
The *cis*-element of *OsSCL30* promoter interacted with OsLHY in *vivo* and *in vitro*. ***A)*** Interaction of OsLHY and *OsSCL30* promoter via Y1H. NBS: pHIS2-3 × AAAAATCT. Negative control: pGADT7-Rec-p53 + pGADT7-OsLHY; Positive control: pGADT7-Rec-p53 + pGADT7-53. B***)*** The vector construction for effector and reporter (Upper) and the *OsSCL30pro^M^:LUC* signal (lower). Mock, the reporter construct and empty effector construct was co-infiltrated into tobacco leaf. Significant differences were identified at 1％(**) probability levels with 3 biological replicates, by Student’S t-test.C***)*** The interaction of OsLHY and *cis*-element of the *OsSCL30* promoter in *vitro*. The fused GST-OsLHY was purified form *E. coli* cells and used for binding assays with probes. The GST protein was used as negative control. For competition, 10, 20 and 50 fold excess competitor probes were mixed with biotin-labelled probes. The mutant probe is 5′-biotin-aaaattgtgccccccccacgcttcaacatt −3′. – and + denote that the corresponding proteins or probes are absent and present, respectively.

Likewise, seven potential transcription factors and their corresponding *cis*- elements were predicted (Table S5). Among these, the transcription factor, *OsLHY* also known as *OsCCA1*, is a circadian rhythm regulator [32]. Subsequently, yeast one-hybrid (Y1H), dual-luciferase reporter (DLuc), and electrophoretic mobility shift assay (EMSA) were carried out to further confirm the prediction in *vivo* and in *vitro*. Firstly, the promoter fragment with predicted *cis*-element, 3 × AAAAATCT, was ligated with the pAbAi vector as bait. The positive bait yeast obtained was subjected to ABA^r^ background expression level detection, and it was found that Y1H Gold was inhibited under 600 ng/mL AbA SD/- Ura medium conditions (Fig. S4). The Y1H results showed that OsLHY interacted with the *cis*-element of *OsSCL30* promoter in *vivo* (Fig. 3A). The DLuc assay present strong signal when proOsSCL30:LUC and pro35S:OsLHY co-expressed compared with the mock. The increased luciferase activity indicated that *OsSCL30* expression was upregulated by transcript factor, OsLHY (Fig. 3B). The EMSA confirmed the interaction of *cis*-element of *OsSCL30* and OsLHY *in vitro* (Fig. 3C). Altogether, in *vivo* and in *vitro* assays demonstrated that OsLHY bound to the *cis*-element (3 × AAAAATCT) of *OsSCL30* and increased the its expression.

### OsSCL30 interacts with DHT1

**Fig. 4:**
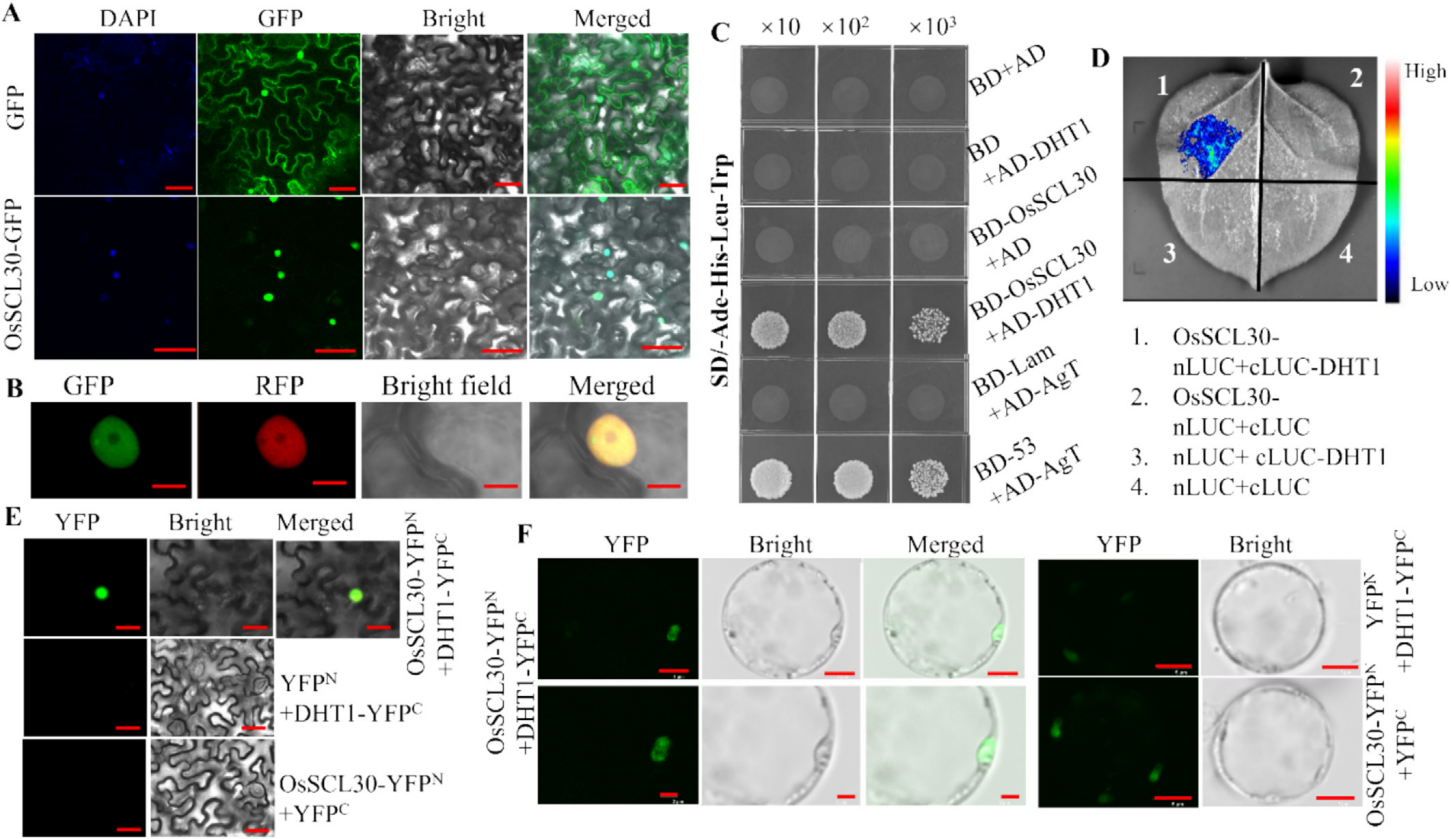
The subcellular localization of OsSCL30 and its interacting with DHT1. A) The subcellular localization of OsSCL30. The OsSCL30 was fused with GFP. DAPI was used as nuclear marker. Scale bar = 50 μm；B) The subcellular localization of OsSCL30. The OsSCL30 was fused with GFP, and the nuclear marker was fused with RFP. Scale bar = 5 μm；C) The interaction of OsSCL30 and DHT1 via Y2H assay. D) The interaction of OsSCL30 and DHT1 via LUC in *vivo*. E) The interaction of OsSCL30 and DHT1 via BiFC in tobacco leave in *vivo*; Scale bar = 20 μm. F) The interaction of OsSCL30 and DHT1 via BiFC in rice protoplasts. The scale bar for OsSCL30-YPF^N^ + DHT1-YFP^C^ lower was 2 μm, the remaining scale bars were 5 μm. Each experiment contained at least 3 independent biological replicates.

We predicted the subcellular localization of OsSCL30 online via the WoLF PSORT (Weblink: https://wolfpsort.hgc.jp), indicating nuclear localization. To confirm it, an OsSCL30-GFP fusion protein was constructed and transiently expressed in tobacco leaves. The 4′,6-diamidino-2-phenylindole (DAPI) was set as nuclear marker to observe GFP signal distribution. The confocal microscopy revealed that the GFP signal of OsSCL30 co-localized completely with DAPI in the nucleus (Fig. 4A) confirming nuclear localization of OsSCL30, consistent with the bioinformatic prediction and previous study, which showed that OsSCL30 was localized in the nucleus of rice [31]. Meanwhile, we transformed OsSCL30-GFP and nuclear marker-RFP fused proteins into *Nicotiana benthamiana* leaves. The green and red fluorescent signals merged in the nucleoplasm but not in the nucleolus (Fig. 4B).

OsSCL30 belongs to the plant-specific SCL subfamily within the SR protein family, containing a serine/arginine (SR)-rich domain (RS domain) and an RNA recognition motif (RRM), which often mediates protein–protein interactions. To identify interacting partners of OsSCL30, a yeast two-hybrid (Y2H) screen was performed using OsSCL30 as bait to detect in a rice cDNA library. Primary screening was conducted on SD/-Leu-Trp-His medium, followed by secondary screening on SD/-Ade-His-Leu-Trp medium, yielding multiple positive clones. The positive clones revealed that one clone encoded DHT1, a member of the RNA binding protein super family, which has previously been shown to localized in the nucleus, and physically interact with U1-70 K and SR proteins including OsSCL30 by co-immunoprecipitation (Co-IP), *in vitro* pull-down assay, and firefly luciferase (LUC) complementation [24]. To validate the interaction further, a direct pairwise Y2H confirmed that OsSCL30 interacted with DHT1 (Fig. 4C). Luciferase Complementation Imaging (LCI) Assays were carried out. The luciferase activity was detected on the leaf epidermal cells where OsSCL30-nLUC and cLUC-DH1was co-expressed, whereas no luciferase activity was detected in the controls. The result showed that OsSCL30 and DHT1 physically interacted with each other (Fig. 4D). Additionally, bimolecular fluorescence complementation (BiFC) assays were performed by co-expressing OsSCL30-YFP^N^ and DHT1-YFP^C^ in tobacco leaf epidermal cells and rice protoplasts (Fig. 4, Fig. 4). Strong YFP fluorescence signals were observed in the nucleus via confocal microscopy (Fig. 4E). No fluorescence was detected in the controls, expressing OsSCL30 or DHT1 alone. The BiFC assays in rice protoplasts confirmed the interaction of OsSCL30-YFP and DHT1, which displayed a distinct speckled pattern within the nucleus and was excluded from the nucleolus (Fig. 4F), which was consistent with the subcellular localization results (Fig. 4, Fig. 4). Overall, these results indicated that OsSCL30 and DHT1 may form a splicing complex in nucleoplasm to regulate RNA splicing or transcriptional regulation.

### OsSCL30 regulate splicing of target genes involved in heading date and plant height

DHT1 have been found to regulate pre-mRNA splicing as a component of spliceosome with U1 snRNP and various SR proteins in rice, including OsSCL30 [24], indicating these splicing factors may assemble into a functional spliceosome complex.

Therefore, RNA-seq was carried out to analyze differentially expressed genes (DEGs) or splicing isoforms between the WT (Nipponbare) and *scl30-1*. The KEGG pathway analysis of DEGs between the WT and *scl30-1* revealed significant enrichment in metabolic and genetic information processing pathways. The 15 enriched pathways included ribosome (map03010), diterpenoid biosynthesis (map00904), starch and sucrose metabolism (map00500), sesquiterpenoid and triterpenoid biosynthesis (map00909), and circadian rhythm-plant (map04712) (Fig. S5; Table S6). Notably, the enrichment of the ribosome pathway is consistent with previous findings in Arabidopsis SC35/SCL mutants [20], suggesting a conserved role of SR proteins in modulating the expression of ribosomal genes across different plant species and genetic backgrounds.

For differentially spliced genes, the KEGG analysis showed predominant enrichment in various metabolic pathways, including cyanoamino acid metabolism (map00460), arachidonic acid metabolism (map00590), fatty acid degradation (map00071), glycine, serine and threonine metabolism (map00260), and starch and sucrose metabolism (map00500) (Fig. S6; Table S7). Notably, we identified alternative splicing events in the *OsFTL8* gene involved in the photoperiod pathway.

**Fig. 5:**
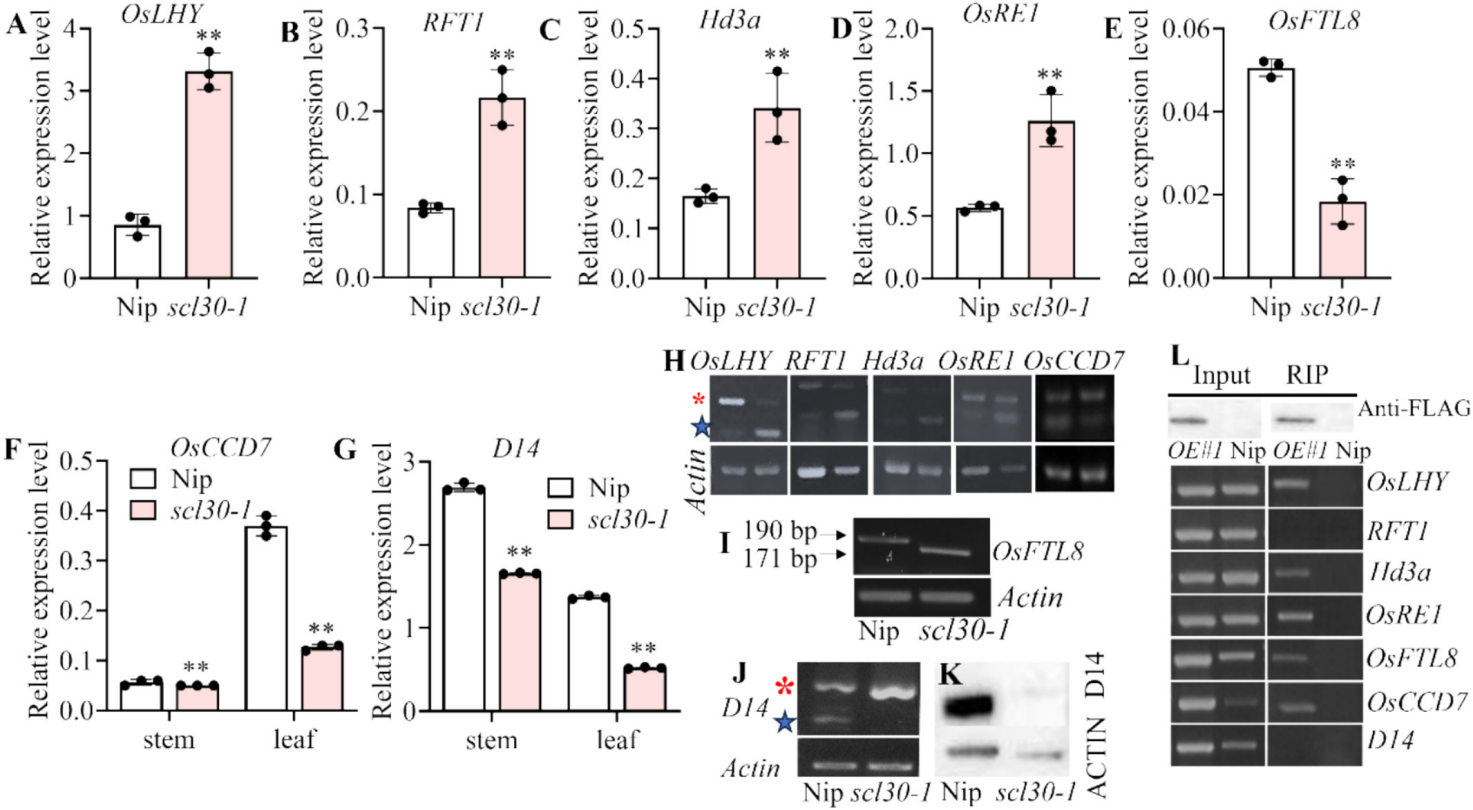
Identification of genes with differentially expression and alternative splicing between the WT (Nipponbare) and gene-editing line, *scl30-1*. ***A***-E***)*** The expression level of the genes relative to heading date. F-G***)*** The expression level of the genes relative to plant height. H) Analysis of constitute splicing effect between Nipponbare and *scl30-1* using RT-PCR. I***)*** The alternative splicing of *OsFTL8* between Nipponbare and *scl30-1*. J) Analysis of alternative splicing effect in the Nipponbare and *scl30-1* using RT-PCR. H-I) The asterisk indicates unspliced pre-mRNAs, while the star indicates spliced mature mRNA. K) The western blotting of D14. L) Confirmation of the target RNAs of OsSCL30 using RIP. The top gels show western blotting of proteins present from crude extracts in Nipponbare, overexpression lines with OsSCL30-flag and proteins immunoprecipitated (IP) with the anti-FLAG antibody. RT-PCR was carried out to detect the association of target genes. The data represent means ± SD of three biological replicates, and ** indicates statistically significant difference at 1 % probability level, as determined by Student’s *t*-test.

Based on phenotype of *scl30* mutants, we focused on circadian rhythm-plant pathways and plant height-related pathways. Notably, bioinformatic analysis of the mis-spliced transcripts did not reveal the introduction of premature termination codons (PTCs), suggesting that the observed reduction in these mature mRNA levels could be a direct consequence of altered splicing efficiency rather than secondary degradation via nonsense-mediated decay (NMD). Therefore, the splicing effect and alternative splicing analysis were carried out. The qRT-PCR analysis revealed significant upregulation of photoperiod-related genes *OsLHY* (LOC_Os08g06110) [33], *RFT1* (LOC_Os06g06300) [34], *Hd3a* (LOC_Os06g06320) [4], and *OsRE1* (LOC_Os01g07880) [35], while *OsFTL8* (LOC_Os01g10590) showed reduced expression (Fig. 5, Fig. 5). In the carotenoid biosynthesis pathway, the dwarfism-related gene *OsCCD7* (also known as *HTD1*) (LOC_Os04g46470), which regulated tillers and plant height [11,12], exhibited obvious downregulation. The qRT-PCR analysis showed reduced *OsCCD7/HTD1* expression in *scl30-1* stems and leaves compared to the WT (Fig. 5F). Although RNA-seq results did not detect expression change in *D14/HTD2*, a gene previously reported to influence plant height through splicing regulation by DHT1 [24], subsequent qRT-PCR revealed the expression significantly decreased in the *scl30-1* mutants (Fig. 5G). To test constitutive and alternative splicing, we first examined the transcription of target genes using RT-PCR and real-time PCR (Fig. 5, Fig. 5, Fig. S8 and S9). Interestingly, we found decreased unspliced pre-mRNA of *OsLHY*, *RFT1*, *Hd3a*, and *OsRE1* but increase accumulation of unspliced pre-RNA of *OsCCD7/HTD1* in the *scl30-1* mutant (Fig. S9B). Correspondingly, the splicing efficiency was higher on *OsLHY*, *RFT1*, *Hd3a*, and *OsRE1*, and lower on *OsCCD7/HTD1* gene in was higher and the *scl30-1* mutant (Fig. S9C). The RT-PCR analysis of the alternative splicing event in *OsFTL8* demonstrated an A3SS (Alternative 3′ Splice Site) event. The WT predominantly expressed pre-A3SS isoforms, whereas *scl30-1* mutants mainly accumulated post-A3SS spliced variants (Fig. 5I).

The western blotting confirmed the D14/HTD2 decreased in the *scl30-1* mutants (Fig. 5K). These findings suggest that both expressions change of photoperiod-related genes and alternative splicing patterns, led to accelerated heading time, while altered expression of plant height-associated genes *OsCCD7/HTD1* and *D14/HTD2* may regulate the height in the *scl30* mutants.

By bioinformatic analysis, we found that OsSCL30 contained an RNA binding domain from 38 to 120 aa (Supplementary Fig. S7), indicating its function on regulating pre-mRNA splicing. RNA immunoprecipitation (RIP) was carried out to verify its binding on the candidate target genes based on the RT-PCR (Fig. 5L). The immunoprecipitated RNAs were reverse-transcripted into cDNAs after they were immunoprecipitated using an anti-FLAG antibody. The RT-PCR results from immunoprecipitated RNAs present that *OsLHY* (also known as *OsCCA1*), *OsRE1*, *OsFTL8 and OsCCD7* could be bound by OsSCL30. These findings indicate that OsSCL30 and DHT1 form a spliceosome to be involved in regulating the splicing effect of these target genes, thereby influencing heading date and plant height.

The OsLHY/OsCCA1 as a transcript factor positively regulates expression of *D14* [36]. We found the expression of *OsLHY* was increased, whereas *D14* expression was decreased. As the target gene of DHT1[24], we speculated that splicing effect of D14 was decreased which was affected by the spliceosome of DHT1 and OsSCL30. Thus, we detected the pre-mRNA of *D14* by RT-PCR and D14 concentration by Western blotting (Fig. 5, Fig. 5). The RT-PCR showed that the unspliced *D14* pre-mRNA increased in the *scl30-1* mutant. However, the matured *D14* mRNA and the D14 concentration in the *scl30-1* mutant decreased, suggesting that the OsSCL30-containing spliceosomal complex was involved in regulating *D14* pre-mRNA splicing.

**Fig. 6:**
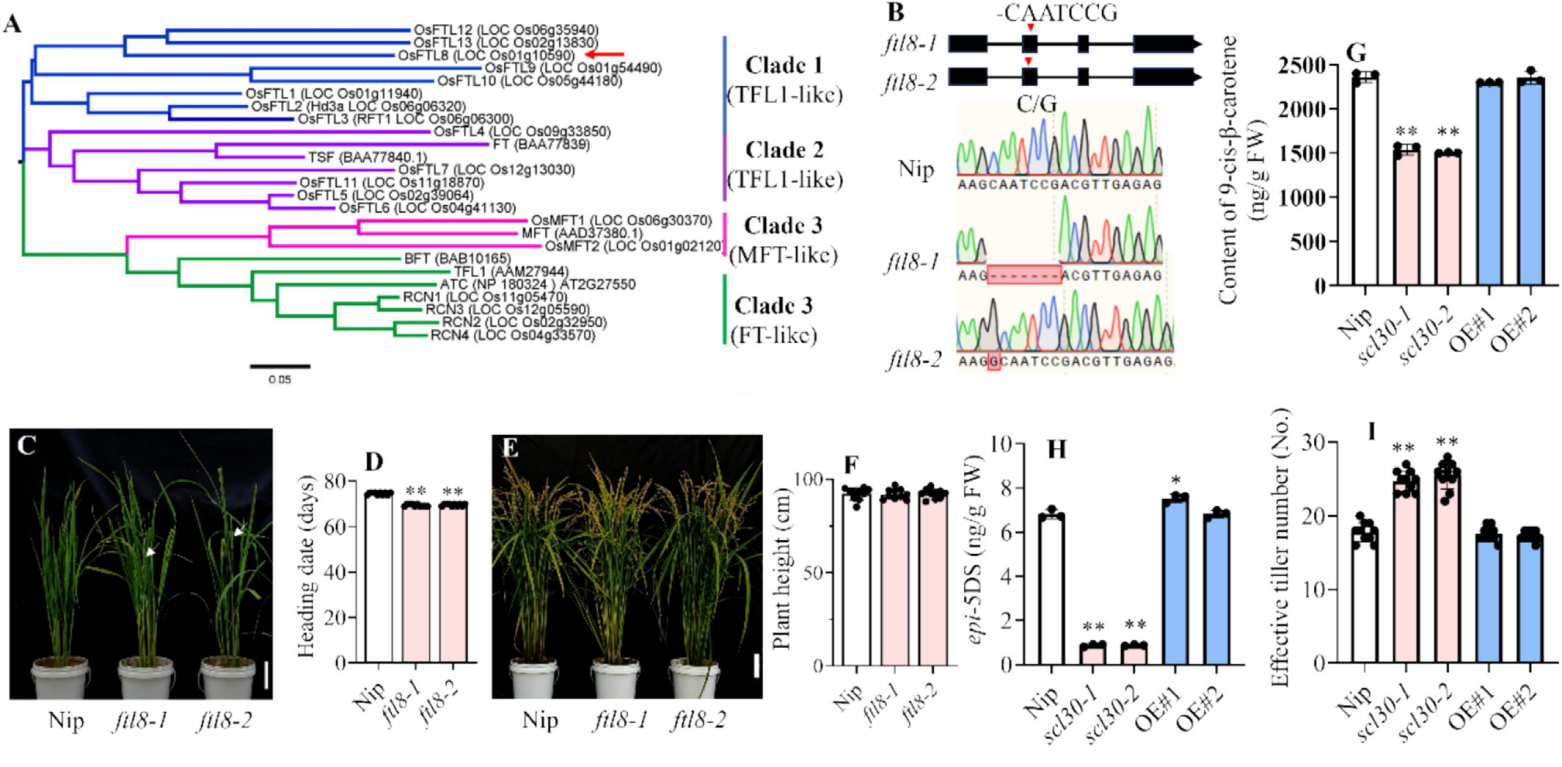
Phylogenetic tree, phenotype of OsFTL8 and the pathway of strigolactone synthesis that *OsSCL30* involved in. A) Phylogenetic tree for the PEBP proteins in *Arabidopsis* and rice. Sequence alignment was performed with MEGA7, and displayed graphically by TreeView. The red arrowhead indicated OsFTL8. B) The mutation sites on two gene-editing lines created by CRISPR/Cas9 system. The unfilled boxes, the filled boxes, lines, and red triangle represent UTR (5′-URT and 3′-UTR), the CDS, introns, and editing sites, respectively. (−) and (/) represent the deletion site, and substitution, respectively. C, D) The phenotype and histogram of heading date between Nip and gene editing lines *ftl8*. The white arrowheads indicated heading panicles. Scale bar = 10 cm. E, F) The phenotype and histogram of plant height between Nip and gene editing lines *ftl8*. The white arrowheads indicated heading panicles. Scale bar = 10 cm. G) The content of 9-*cis*-β-carotene. H) epi-5DS. I) Effective tiller number. Nip:Nipponbare；*ftl8-1, ftl8-2*: gene-editing lines of *OsFTL8*. ** indicates the significantly difference compared to the Nipponbare at 1 % level, as determined by Student’s *t*-test. The data represent means ± SD (10 replicates for panels D, F and I; three biological replicates for panels G and H).

In the photoperiod-related signaling pathways, all genes except *OsFTL8* in the current study were previously identified to regulate heading date. According to the annotation of Rice Genome Annotation Project, *OsFTL8* is an FT-Like8 homologous to Flowering Locus T gene, which belong to PEBP (for phosphatidylethanolamine-binding protein) family. To characterize the function of *OsFTL8*, we conduct *in-silico* screening against an in-house sequence database of gene libraries in *Arabidopsis* and rice. The phylogenetic analysis of OsFTL8 and its homologues in rice, and *Arabidopsis* presented three major clades. TFL1-like proteins covered the clade 1 and clade 2. The clade 3 can be divided into two branches, MFT-like and FT-like proteins (Fig. 6A). The sequence alignment of TFL1-like proteins revealed highly conserved, indicating their function similarity (Fig. S10). Among these 13 proteins, the proteins which have been characterized were involved in the heading date, except that *OsFTL5*, *OsFTL6*, *OsFTL7*, *OsFTL9*, *OsFTL11* and *OsFTL13* have not been cloned. *OsFTL1*, *OsFTL3* and *OsFTL10* induced heading, while *OsFTL12*, *OsFTL4* and *OsFTL6* suppressed heading in rice [[37], [38], [39], [40]]. The allele at the *OsFTL2*/Hd3a locus from Kasalath promotes heading under SD conditions, whereas it delayed heading under LD conditions [41].

To functionally characterize *OsFTL8*, we generated knockout mutant lines (*ftl8-1* and *ftl8-2*) by CRISPR/Cas9 technology. The *ftl8-1* line exhibited a 7-bp deletion (CAATCCG) starting at nucleotide position 237 downstream of the ATG initiation codon, resulting in a premature termination codon at position 243, while *ftl8-2* carried a single guanine (G) insertion at position 238, leading to an earlier stop codon at position 297 (Fig. 6B). Phenotypic analysis revealed that both knocked-out mutants showed significantly earlier heading dates by 5–6 days compared to the WT (Nipponbare) (Fig. 6, Fig. 6). No difference in plant height was observed between the WT and *ftl8* lines (Fig. 6, Fig. 6). These findings strongly suggest that *OsFTL8* functioned as a novel regulator in the heading date.

One of plant height-associated genes, *OsCCD7* (also known as *HTD1*), encodes the carotenoid lyase, which is one of the important enzymes involved in the strigolactone biosynthesis using the 9-*cis-*β-carotene as its substrate [42]. Thus, we measured the content of 9-*cis*-β-carotene among the WT, *scl30* knocked-out lines and overexpression lines, and found that the content of *scl30* knocked-out lines was significantly decreased than the WT and overexpression lines, indicating low expression of *OsCCD7*/*HTD1* resulted in the degradation of 9-*cis*-β-carotene (Fig. 6G). *D14* (also known as *HTD2*) encodes an esterase that is a component of the strigolactone signaling and functions downstream of strigolactone (SL) synthesis [43]. Both *OsCCD7/HTD1* and *D14/HTD2* were involved in the SL pathway and negatively regulates rice tiller number, we detected these two indices. The *scl30* knocked-out lines accumulated significantly lower levels of *2*′*-epi*-5-deoxystrigol, leading to more tiller number and decreased plant height compared with the WT and overexpression lines (Fig. 6, Fig. 6 and Fig. 1E. 1F). From the above results, it can be inferred that *OsCCD7/HTD1* and *D14/HTD2* played a potential role in the tiller number, plant height by SL pathway.

### The photoperiod effect on the expression of the rhythmic genes

**Fig. 7:**
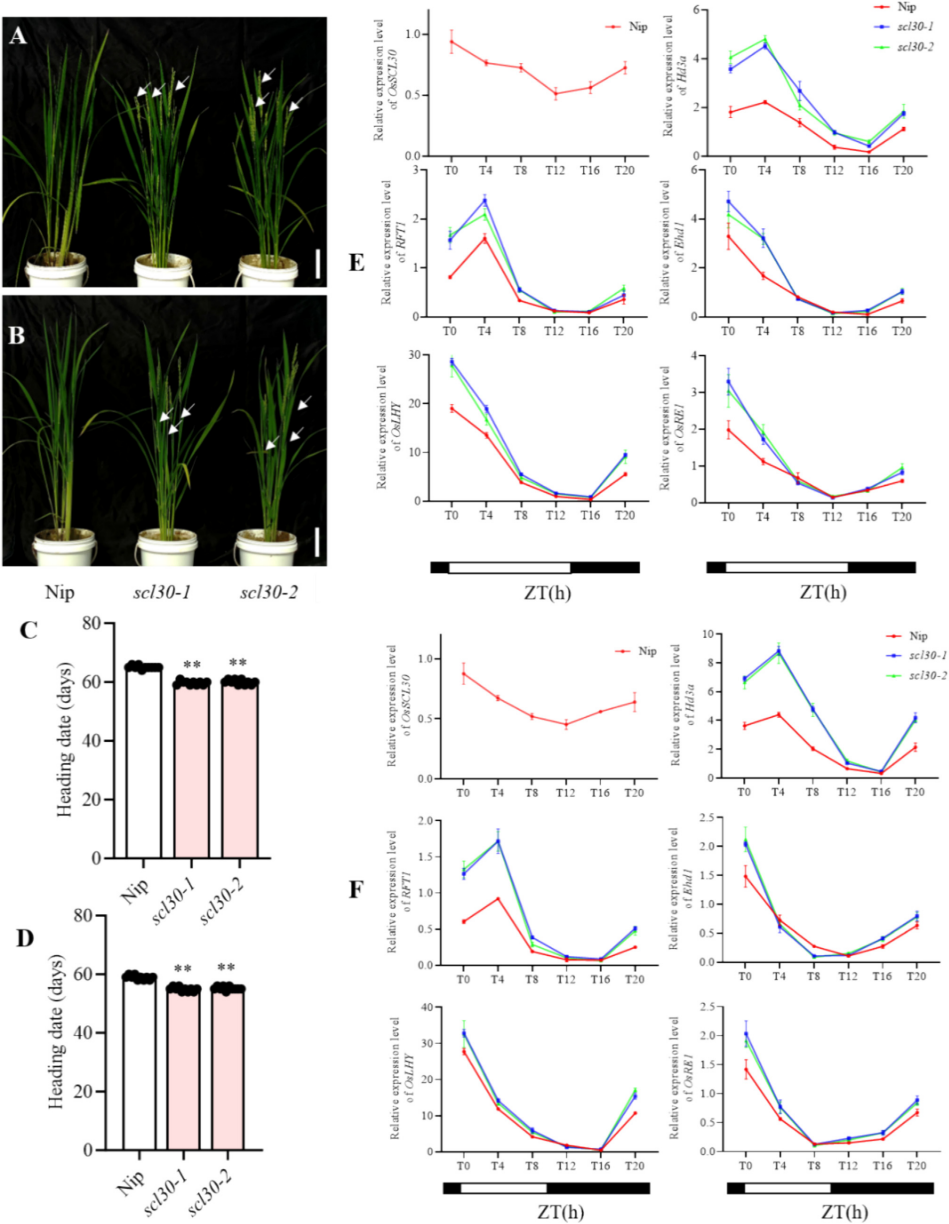
Comparison of heading date and expression of photoperiod-related genes between Nip, gene editing lines *scl30-1* and *scl30-2* under CLD and CSD conditions. A-D) Comparison of heading date between Nip, gene editing lines *scl30-1* and *scl30-2* under CLD (A, C) and CSD (B, D) conditions. The arrowheads indicated the panicles. The data represent means ± SD with 10 replicates. ** indicate significant differences at *p* ≤ 0.01, as determined by Student's *t*-test. E-F) Relative transcription levels of *OsSCL30*, *Hd3a*, *RFT1*, *Ehd1*, *OsLHY* and *OsRE1* gene under CLD (E) and CSD (F) condition. The red circle, blue square and green triangle represented Nip, *scl30-1* and *scl30-2*, respectively. Leaf samples were collected every 4 h from Niponbare, gene-editing line *scl30-1* and *scl30-2* plants at 45-days age seedling. ZT, Zeitgeber time. The black and white boxes indicated the dark and light periods, respectively. *OsActin* was used as reference control, and its expression level was detected by qRT-PCR. Data were expressed as the mean ± SD of three biological replicates.

The WT and knocked out lines, *scl30-1* and *scl30-2*, were subjected to controlled photoperiod conditions under control long-day (CLD: 14 h light/10 h dark) and control short-day (CSD: 10 h light/14 h dark) regimes. Compared with the WT, both *scl30-1* and *scl30-2* mutants exhibited significantly earlier heading dates by approximately 5–6 days under CLD conditions (Fig. 7, Fig. 7) and 4–5 days under CSD conditions (Fig. 7, Fig. 7B-D).

To elucidate the regulatory relationship between *OsSCL30* and key heading time determinants, we analyzed the expression patterns of major heading-related genes under both photoperiod conditions. *OsSCL30* displayed rhythmic expression under both CLD and CSD conditions, peaking at dawn followed by gradual downregulation during light periods, and reaching minimal levels at dusk with subsequent upregulation during dark phases (Fig. 7, Fig. 7).

Under CLD conditions, the florigen genes, *Hd3a* and *RFT1*, showed significantly elevated mRNA levels in *scl30* mutants compared to the WT plants (Fig. 7, Fig. 7). To investigate whether *OsSCL30* regulates *Hd3a/RFT1* through Ehd1-mediated pathways, we examined *Ehd1* expression and observed marked upregulation in both mutants. Similar expression patterns for *Hd3a*, *RFT1*, *Ehd1*, *OsLHY*, and *OsRE1* were observed under CSD conditions (Fig. 7, Fig. 7). These findings suggest that *OsSCL30* regulates heading date through the Ghd7-Ehd1-Hd3a/RFT1 signaling pathway.

### Natural Variation of OsSCL30 regulating heading date and plant height

**Fig. 8:**
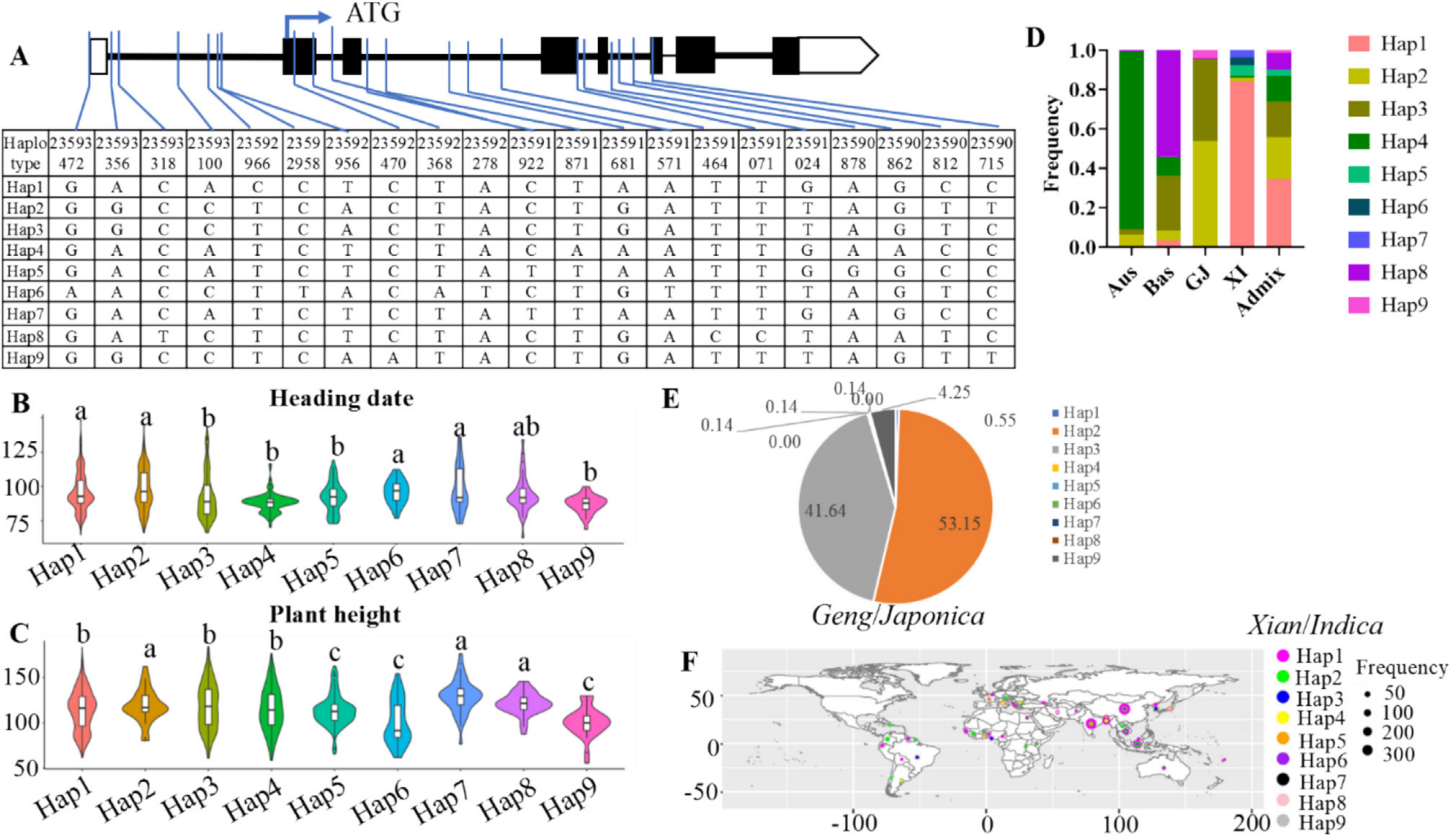
Genetic difference and association analysis of *OsSCL30*. A) Analysis of major 9 haplotypes of the *OsSCL30* from 2475 Asian cultivated rice from the 3000 Rice Genomes Project (3 K RGP). B, C) Statistical analysis of heading data (B) and plant height (C) of 9 main haplotypes. Different letters above error bars indicate significant differences among different haplotypes at *p* ≤ 0.05, as determined by one-way ANOVA analysis. D) Frequency of the 9 haplotypes among 5 subpoluations of *Aus*, *Bas*, *Geng*/*Japonica* (*GJ*)*, Xian*/*Indica* (*XI*) and *Admix*. E) Distribution ratio of the 9 haplotypes between *Geng*/*Japonica* and *Xian*/*Indica*. F) Geographical distribution of these 9 haplotypes.

According to the OsSCL30 function on heading date and plant height, we aimed to explore favorable *OsSCL30* haplotype. A haplotype analysis based on the SNPs in the CDS of *OsSCL30* genome from 277 cultivated rice accessions was conducted from the public data [44]. Totally, there were 21 SNPs, grouping 14 haplotypes (Fig. S11). We chose the main haplotypes (hap1-hap9) with frequencies *>* 0.6 % for further analysis (Fig. 8A). These 9 haplotypes presented significant variation in heading date and plant height (Fig. 8, Fig. 8)*.* The accessions from hap1, hap2, hap 6 and 7 showed late heading date, whereas hap3, hap4, and hap5 presented early heading date (Fig. 8B). For the trait of plant height, these 9 haplotypes were divided into three groups (Fig. 8C). *OsSCL30* hap7, hap8 and hap2 accessions displayed highest plant height, while plant height of hap5, hap6 and hap9 was shortest. The remaining accessions from hap1, hap3 and hap4 were located in the middle. The results indicated that hap2 and hap7 contributed to late heading date and high plant height, while hap5 and hap9 led to early heading date and short plant height. According to the population structure of 3,010 diverse accessions from Asian cultivated rice [45], we found hap1, hap 2 and hap5 predominantly occupied *Xian*/*Indica* (*XI*), G*eng*/*Japonica* (*GJ*) and Aus, respectively (Fig. 8D). The hap2 and hap3 accounted for 94.79 % of G*eng*/*Japonica* rice, while *Xian*/*Indica* type harbored hap1 (84.2 %) and hap4 (5.28 %) (Fig. 8E). Among these hapotypes, hap1 and hap2 were distributed across multiple regions and exhibited broadly similar geographic patterns. The hap3 and hap4 were primarily found in outh Asia and Southeast Asia. The Hap5 was predominantly distributed in tropical and subtropical regions, while hap6 was mainly localized to tropical areas. The hap7, hap8, and hap9 were largely confined to East Asia (Fig. 8F). The results indicated distribution imbalance of *OsSCL30* haplotypes.

## Discussion

### OsSCL30 participates in heading date by Ehd1-Hd3a-RFT1 pathway

Under natural long-day conditions, the gene-edited lines *scl30-1* and *scl30-2* exhibited a heading date 5–6 days earlier than Nipponbare, while the overexpression lines showed heading date delayed by 8–11 days compared to the *scl30-1* (Fig. 1). Under both LD and SD conditions, the heading dates of *scl30-1* and *scl30-2* were 5–6 days and 4–5 days earlier than Nipponbare, respectively (Fig. 7). These results indicated that *OsSCL30* regulated rice heading date under both photoperiod conditions and functioned as a negative regulator of flowering.

In this study, we observed increased expression levels of photoperiod-related genes *OsLHY*, *OsRE1*, *Ehd1*, *Hd3a*, and *RFT1* in the gene-edited mutant lines, while *OsFTL8* expression decreased (Fig. 5, Fig. 7, Fig. 7). Under both long-day and short-day conditions, elevated transcriptional levels of *OsLHY* and *OsRE1* promoted *Ehd1* expression, ultimately enhancing the expression of *RFT1* and *Hd3a*, which were florigen genes (Fig. 7), leading to accelerated heading.

*OsLHY*/*OsCCA1* is the ortholog of *AtCCA1* and *AtLHY* in *Arabidopsis* [46]. *OsLHY*/*OsCCA*1 is a flowering inducing factor, and previous studies have shown that the *OsCCA1-OsPRR37*/*DTH8-Ehd1* module is a unique pathway for regulating the expression of rice anthocyanins *Hd3a* and *RFT1*. The loss of OsCCA1a function led to increased expression of the floral repressors OsPRR37 and DTH8, thus repressing Ehd1–Hd3a–RFT1 pathway [32]. *OsRE1* is a secondary effector gene that regulates heading date. OsRE1, as a transcription factor, can bind to the A-box element on the Ehd1 promoter to micro regulate the expression of *Ehd1*, thereby micro regulating the heading date in rice [35].

*OsFTL8* was a novel gene characterized in the current study. Phylogenetic analysis and alignment of OsFTL8 with its homologues revealed highly conserved, indicating their potential function similarity. In our study, the mutation of *OsFTL8* led to early heading date (Fig. 6, Fig. 6), a finding consistent with the known roles of other identified *OsFTL* genes [[37], [38], [39], [40], [41]]. These results suggest that *OsFTL8* may potentially function as a novel regulator influencing heading date; however, further genetic evidence, such as the analysis of overexpression lines, is required to confirm its precise biological role.

Temporal expression profiling under altered photoperiod conditions further strengthened the causal directionality of the OsLHY-OsSCL30 feedback loop (Fig. 7, Fig. 7D, E). The results clearly show that *OsSCL30* expression exhibits a robust diurnal rhythm, peaking at dawn, which is consistent with its direct transcriptional activation by OsLHY (Fig. 3). Conversely, in the *scl30* mutants, the expression amplitude of *OsLHY* itself was significantly altered, demonstrating that OsSCL30 is required for fine-tuning *OsLHY* oscillation. This reciprocal regulation, observed in rice plants under both long-day and short-day conditions, provides strong in *vivo* evidence for a functional feedback loop between *OsLHY* and *OsSCL30*. This feedback modulation of OsLHY by OsSCL30 likely contributes to the broader changes in the circadian network that we observed. For instance, the expression changes of *OsLHY* in the mutants may affect rice heading by regulating downstream targets such as *OsGI* [33].

In rice, OsLHY-OsGI-Hd1 is a conserved pathway for regulating heading stage [33]. Moreover, *OsGI* is not the only target of OsLHY, and multiple targets may jointly regulate the heading stage of rice. For example, DTH8 interacts with Hd1 to inhibit *Hd3a* expression by altering the level of H3K27me3 in its promoter [47]. Meanwhile, Hd1, Ghd7, and DTH8 can form protein complexes to function on heading date [48]. OsRE1 inhibited the expression of *Ehd1* by interacting with DTH8 directly. Here, OsRE1 may act as a bridge between *Ehd1* and *DTH8* to regulate rice heading stage [49]. Therefore, beyond its role in splicing, OsSCL30 is embedded in the circadian clock network through its feedback interaction with OsLHY, thereby affecting rice heading date through a complex photoperiod regulatory network. While our study firmly establishes the role of OsSCL30 in regulating the transcription of core florigen genes, future research exploring the subsequent dynamics of florigen transport and activation in the shoot apical meristem will provide an even more comprehensive understanding of this flowering pathway.

### OsSCL30 is involved in plant height by regulating strigolactone biosynthesis

In this study, compared with the WT (Nipponbare), the gene edited lines *scl30-1* and *scl30-2* showed a dwarf phenotype, while the overexpression line showed a tall phenotype compared to the gene edited line s*cl30-1* (Fig. 1). The expression levels of *OsCCD7*/*HTD1* and *D14*/*HTD2* genes were detected to be reduced in the gene editing line *scl30-1* by qRT-PCR (Fig. 5). *OsCCD7* encodes carotenoid lyase dioxygenase, which is one of the important enzymes during the biosynthesis of strigolactone. Its mutant exhibits multiple tillering and dwarfing traits [11,50,51]. During the synthesis of strigolactones (SLs), 9-*cis-*β-carotene is cleaved by OsCCD7 to form 9-*cis*-β-apore-10′-carotene aldehyde [12]. DHT1 directly binds to the pre-mRNA of *D14* gene, regulating the splicing of *D14*. D14 may act as an enzyme for the transformation of strigolactone into its active form, downstream of SL synthesis [43]. Compared with the wild type, the *scl30-*1 mutant accumulated lower concentrations of 2-deoxystrigol (2 '- epi-5-deoxystrigol) (Fig. 6). It is speculated that the *OsSCL30* gene may regulate rice plant height by affecting the expression levels of the genes related to strigolactone biosynthesis. *OsSCL30* was involved in the regulation of rice plant height through multi-level mechanisms, including OsLHY, which might participate in transcriptional regulation of *OsSCL30*, and DHT1, which acts with OsSCL30 to form a splicing complex.

The OsLHY-OsSCL30-DHT1 regulatory module uncovered here provides novel mechanistic insights into how pre-mRNA splicing influences agronomic traits. To contextualize our findings within cereal systems, we further discussed conservation and related splicing mechanisms in other crops. Synteny analysis has revealed numerous orthologs of rice SR genes in other cereals, with 20, 40, and 64 orthologous pairs identified between rice and sorghum, maize, and wheat, respectively [18]. In maize, 27 SR genes have been identified bioinformatically, many of which exhibit tissue-specific and genotype-dependent expression under drought stress [52]. Furthermore, the maize ROUGH ENDOSPERM3 (RGH3) gene, an ortholog of the human splicing factor ZRSR2, is essential for proper endosperm development, its mutation leads to aberrant splicing and developmental defects [53]. In wheat and *Brachypodium distachyon*, 40 *TaSR* and 16 *BdSR* genes were identified and classified into seven subfamilies [54]. Notably, TaSR30 was recently shown to interact with TaASR2L, enhancing the expression of pathogen-related genes and improving resistance to stripe rust [55]. Despite these advances, functional studies on splicing regulators in cereals remain limited, particularly those linking circadian regulation with pre-mRNA processing. Our work helps address this gap by elucidating a splicing regulatory module that contributes to a key agronomic trait in rice.

### OsSCL30-DHT1 splicing complex orchestrates post-transcriptional regulation

Beyond its feedback interaction with OsLHY, the OsSCL30-DHT1 splicing complex serves as a central regulator of post-transcriptional networks, fine-tuning key agronomic traits such as heading date and plant height. Our data demonstrate that OsSCL30 directly binds to multiple flowering-time and plant architecture-related genes and modulates their splicing efficiency, while DHT1 physically associates with D14, a key component in strigolactone signaling, to regulate its alternative splicing patterns. This dual targeting mechanism suggests that the OsSCL30-DHT1 complex integrates endogenous developmental cues (via OsSCL30 targets) and hormonal signals (through DHT1-D14 interaction) to coordinate rice growth and phase transition.

Notably, the splicing targets of OsSCL30 include conserved regulators of photoperiodic flowering (e.g., *Hd1*, *Ehd1* homologs) and *strigolactone*-responsive height control genes. The simultaneous regulation of these targets implies that the splicing complex may synchronize vegetative and reproductive development by generating functionally distinct mRNA isoforms in response to environmental or physiological changes.

The DHT1-D14 interaction adds another regulatory layer. Given that D14 functions as a strigolactone receptor controlling tiller angle and branching, DHT1-mediated splicing of *D14* transcripts might generate protein variants with altered hormone sensitivity. This could explain observed pleiotropic effects on both heading date (through crosstalk with florigen pathways) and plant height (via potential modulation of strigolactone-mediated internode elongation).

Evolutionarily, the expansion of splicing factors in cereals (e.g., 40 % more SR proteins in rice than *Arabidopsis*) suggests enhanced post-transcriptional regulatory capacity for environmental adaptation. Our identification of OsSCL30-DHT1 targets provides experimental support for this hypothesis, revealing how splicing plasticity contributes to trait diversification in crops. Future studies should explore whether genetic manipulation of this complex can uncouple its effects on flowering time and plant architecture, a potential strategy for breeding climate-resilient rice varieties.

In conclusion, the OsSCL30-DHT1 module exemplifies how a single splicing complex can canalize multiple developmental pathways through target-specific RNA processing directly and indirectly. Its dual regulation of photoperiodic flowering genes and strigolactone signaling components positions it as a molecular hub linking environmental cues with morphological adaptation.

In this study, we have identified key target genes through which OsSCL30 could regulate heading date and plant height in rice. Our RNAseq, RT-PCR and RIP-qPCR data provided strong evidence for the association of OsSCL30 with these transcripts. However, the molecular mechanism underlying the binding specificity of OsSCL30 remains an open question. Future work should be directed towards precisely mapping the RNA-protein interaction sites. Techniques such as iCLIP (individual-nucleotide resolution UV crosslinking and immunoprecipitation) would be invaluable to pinpoint the exact binding locations of OsSCL30 on its target RNAs at a nucleotide level. Subsequently, motif enrichment analysis of the high-confidence binding sites obtained from iCLIP could help identify the specific RNA sequence or structural motifs recognized by the RNA recognition motif (RRM) of OsSCL30. This would not only solidify our understanding of its binding preferences but also allow for the genome-wide prediction of novel targets. Furthermore, integrating these precise binding maps with the functional genetic data from our *scl30* mutant could provide a more comprehensive model of how OsSCL30 orchestrates splicing networks to control crucial agronomic traits.

### Interlocked transcriptional and post-transcriptional feedback between OsLHY and OsSCL30 coordinates gene expression in rice

The intricate regulation of gene expression in plants often involves a complex interplay between transcriptional and post-transcriptional mechanisms. In the current study, OsLHY/ OsCCA1 bound to the *cis*-element of *OsSCL30* promoter, enhancing its expression (Fig. 3), while OsSCL30, in turn, interacted with DHT1 to form a splicing complex that modulated the splicing efficiency of *OsLHY* transcripts (Fig. 5A). The transcriptional activation of *OsSCL30* by OsLHY underscored the role of transcription factors in coordinating downstream regulatory pathways (Fig. 5, Fig. 5). Given that OsLHY/ OsCCA1 is a known circadian regulator [33], its direct promotion of *OsSCL30* expression may link circadian rhythms with RNA processing, offering a potential mechanism for diurnal oscillations in splicing efficiency [56]. OsCCA1 can bind to the promoter of flowering inhibitory factors, *DTH8* and *OsPRR37*. The loss of *OsCCA1a* function induces the expression level of *DTH8* and *OsPRR37*, therefore inhibiting the Ehd1-Hd3a-RFT1 pathway [32]. OsCCA1 acts on the upstream of *OsTB1*/*FC1* and dwarf gene *D14*, positively regulating their expression and regulating the SLs pathway [36]. Therefore, OsLHY was involved not only in the heading time, but also in plant height. Conversely, the feedback regulation via OsSCL30-DHT1-mediated splicing of *OsLHY* introduced an additional layer, possibly fine-tuning *OsLHY* splicing efficiency. Moreover, the temporal expression profiling under altered circadian conditions were presented in Fig. 7, Fig. 7. The diurnal expression analyses under both controlled long-day (CLD) and short-day (CSD) conditions in the WT and *OsSCL30 *knockout mutants demonstrated a clear rhythmic expression pattern of *OsSCL30*, with peaks at dawn and troughs at dusk, supporting its circadian regulation. Our study reveals a sophisticated feedback regulatory loop between the transcription factor OsLHY and the splicing-related protein OsSCL30, highlighting the intricate interplay between transcriptional and post-transcriptional regulation in rice. This reciprocal regulation suggests a finely balanced mechanism that ensures precise gene expression control at transcript and post-transcript levels.

In summary, the OsLHY-OsSCL30-DHT1 axis exemplifies how transcriptional and post-transcriptional processes are interconnected to maintain homeostasis and adaptability in rice. This study provides a foundation for further exploration of multilayer gene regulatory networks in rice.

### Divergent but interconnected roles of OsSCL26 and OsSCL30 in regulation via splicing network

As members of the SR protein family, OsSCL26 and OsSCL30 play both distinct and partially interconnected roles in regulating rice development and stress responses. Previous studies have indicated that some SR genes often respond similarly to certain hormonal and environmental cues; for instance, multiple *OsSR* genes showed coordinated expression changes under ABA, GA, drought, salt, and temperature stresses [18]. For exmple, three SR proteins, including *SR40, SCL57*, and *SCL25*, have been implicated in phosphorus uptake and remobilization [57], highlighting the functional diversity within this family. While OsSCL30 has been linked to drought, low temperature, and salt tolerance through modulating reactive oxygen species accumulation [31], OsSCL26 regulates phosphorus homeostasis via transcriptional and post-transcriptional control of phosphate transporters [58]. Nevertheless, the functional relationship between OsSCL26 and OsSCL30 remained unclear. In this study, we demonstrate that loss of *OsSCL26* affects the splicing efficiency of *OsSCL30* (Fig. 1, Fig. 1), suggesting cross-regulation within the SR family. This observation is consistent with documented autoregulatory and compensatory mechanisms among splicing regulators in both *Arabidopsis* and rice [59,60]. Notably, despite both influencing heading date, OsSCL26 and OsSCL30 play non-redundant roles in distinct physiological processes: *OsSCL26* modulates grain morphology and carbohydrate metabolism without affecting plant height [25], whereas *OsSCL30* specifically regulates plant height and heading date (Fig. 1, Fig. 1). Their expression patterns diverge across tissues and in response to hormones and stresses [18], further supporting functional specification. Through transcriptomic and RIP analyses, we found that OsSCL30 directly binds and modulates the splicing of key photoperiodic flowering genes (e.g., *OsLHY*, *RFT1*, *OsFTL8*) and strigolactone signaling components (e.g., *OsCCD7*, *D14*), supporting its role in specific developmental pathways (Fig. 5 and Fig. 6).

Although some functional overlap may exist within the SR protein family, the pronounced phenotypic differences between *osscl26* and *scl30* mutants, combined with the absence of strong compensatory expression changes, argue against full redundancy. Instead, we propose that OsSCL26 and OsSCL30 operate in parallel, each fine-tuning specific aspects of development and stress adaptation, thereby ensuring robust regulation through a diversified splicing network.

**Fig. 9:**
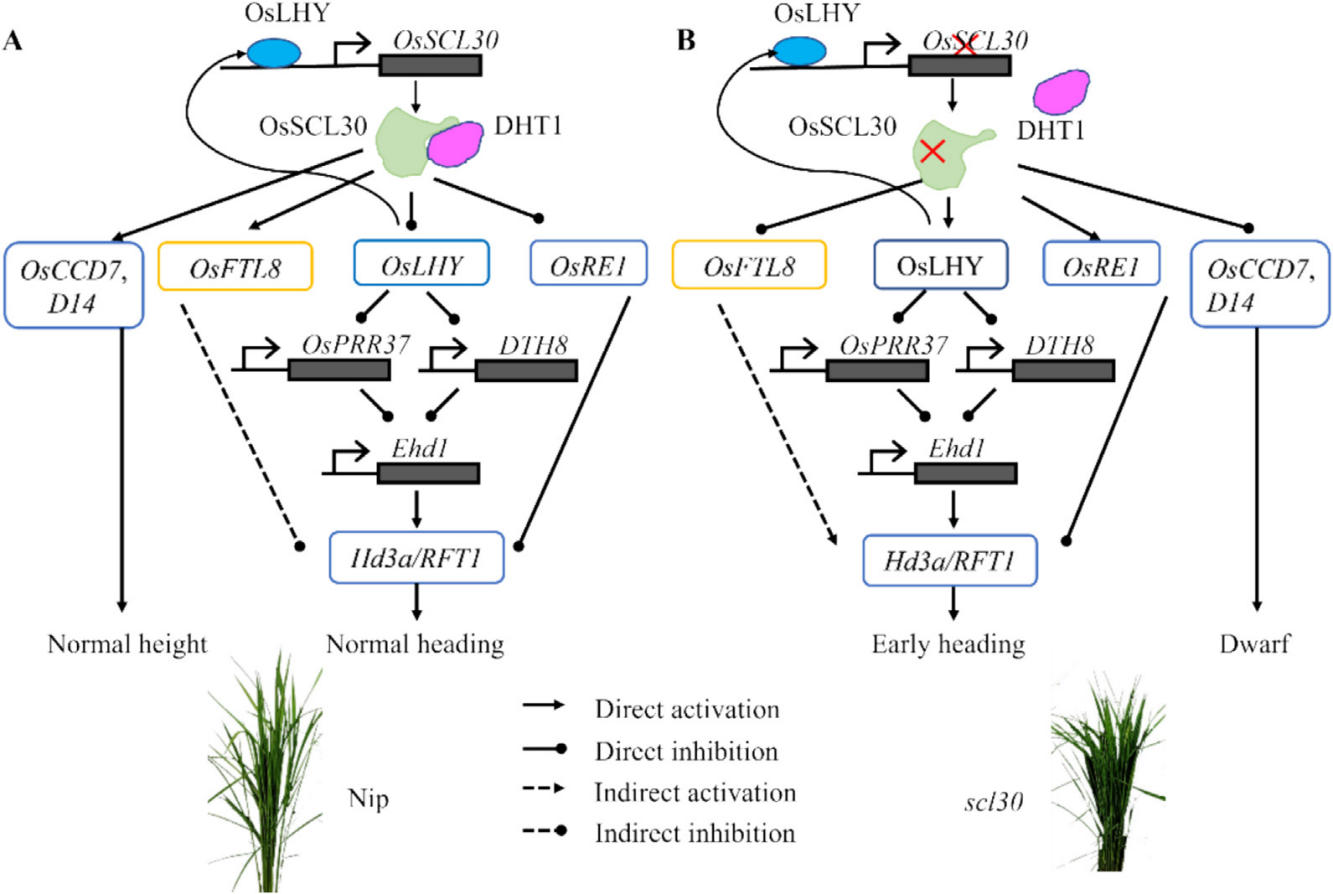
The schematic model for OsSCL30 influence on heading date and height in rice. A) The work model for OsSCL30 involved in heading date and height in rice. OsLHY bound directly the promoter of *OsSCL30* to induce its expression. During pre-mRNA splicing, OsSCL30 interacted with DTH1 to form a spliceosome to affect the transcript and splicing of target genes. Here, the splicing efficiency of *OsLHY* was regulated by OsSCL30 and DTH1 complex. Therefore, a sophisticated feedback loop between the transcription factor OsLHY and the splicing factor OsSCL30 was built. B) In gene editing line *scl30*, mutation of *OsSCL30* leaded to increased expression of *OsLHY* and *OsRE1*. *OsLHY* inhibited expression by binding to promoter region of *OsPRR37* or *DTH8*, thereby activating expression of *Ehd1*. OsRE1 binds to the promoter of *Ehd1* directly to regulate *Ehd1* expression negatively, and *Ehd1* promotes the expression of Florigen *Hd3a* and *RFT1*, leading to early heading. In addition, *OsFTL8* may be involved in the regulation of *Hd3a* and *RFT1*, and other unknown genes may also contribute to the regulation of heading date in rice. Therefore, *OsSCL30* affected the heading date by being involved in the specific flowering pathway, *Ghd7-Ehd1-Hd3a/RFT1*. Meanwhile, OsSCL30-DHT1 complex regulated plant height via SL pathway.

In conclusion, we proposed a schematic model to illustrate how OsSCL30 regulated heading date and plant height in rice multiple rice traits (Fig. 9 and Fig. S12). In this model, OsLHY bound directly the promoter of *OsSCL30* to induce its expression. During pre-mRNA splicing, OsSCL30 interacted with DTH1 to form a spliceosome to affect the transcript and splicing of target genes. Here, the splicing efficiency of OsLHY was regulated by OsSCL30 and DTH1 complex. Therefore, a sophisticated feedback loop between the transcription factor OsLHY and the splicing factor OsSCL30 was build. In gene editing line *scl30*, mutation of *OsSCL30* led to increased expression of *OsLHY* and *OsRE1*. *OsLHY* inhibited the expression of *OsPRR37* or *DTH8* via binding to their promoters, thereby activating expression of *Ehd1*. OsRE1 directly binds to the *Ehd1* promoter to negatively regulate the expression of *Ehd1*, and *Ehd1* promotes the expression of Florigen *Hd3a* and *RFT1*, leading to early heading. In addition, *OsFTL8* may be involved in the regulation of *Hd3a* and *RFT1*, and other unknown genes may also contribute to the regulation of heading date. Therefore, *OsSCL30* affected the heading date of rice by regulating the specific flowering pathway *Ghd7-Ehd1-Hd3a/RFT1*. Meanwhile, OsSCL30-DHT1 complex regulated plant height via SL pathway.

## Conclusions

In the study, we demonstrated that the splicing factor OsSCL30, in coordination with DHT1, formed a functional spliceosomal component essential for regulating heading date and plant height in rice. The OsSCL30 exerted its pleiotropic effects by directly and indirectly modulating the splicing efficiency of specific target genes. Heading date was primarily regulated through the Ghd7–Ehd1–Hd3a/RFT1 pathway, while plant height was controlled via potential strigolactone signaling pathway. *OsSCL30* expression was directly activated by the transcription factor OsLHY, which bound to the *OsSCL30* promoter. Furthermore, OsLHY and OsSCL30 formed a transcriptional-splicing feedback loop, suggesting a mechanism for maintaining regulatory balance in these critical developmental processes. This work elucidated a detailed molecular mechanism by which the spliceosome, specifically through the OsSCL30-DHT1 complex, fine-tunes key agronomic traits for environmental adaptation and yield potential. It established a crucial theoretical foundation for understanding post-transcriptional regulation in rice development, and provided valuable germplasm resources to support future efforts in enhancing rice climate resilience and productivity through genetic breeding programs.

## Compliance with Ethics Requirements

This article does not contain any studies with human or animal subjects.

## Declaration of competing interest

The authors declare that they have no known competing financial interests or personal relationships that could have appeared to influence the work reported in this paper.
