# Supplementary material for: The OsLHY-OsSCL30/OsDHT1 regulatory cascade coordinates heading date and plant height through Pre-mRNA splicing modulation in rice
Source: J Adv Res. 2025 Nov 14;86:87–102. doi: 10.1016/j.jare.2025.11.023 (PMC13453859; doi:10.1016/j.jare.2025.11.023)
Supplement: Supplementary file 1 [file mmc1.docx]

## Supporting Information

# Supplementary tables

## Table S1 Primers used in this study

| Primers | Sequences (5’→3’) |
| --- | --- |
| CRISPR/Cas9 | |
| *SCL30*-gRT1 | GAGTAGTCATCTCGCCGTCTgttttagagctagaaat |
| *SCL30*-OsU3T1 | AGACGGCGAGATGACTACTCTgccacggatcatctgc |
| *SCL30*-gRT2 | GAGGGGATCCATCGCTGTCgttttagagctagaaat |
| *SCL30*-OsU6aT2 | GACAGCGATGGATCCCCTCCggcagccaagccagca |
| U-F | CTCCGTTTTACCTGTGGAATCG |
| gRNA-R | CGGAGGAAAATTCCATCCAC |
| Pps-R | TTCAGAGGTCTCTACCGACTAGTATGGAATCGGCAGCAAAGG |
| Pgs-2 | AGCGTGGGTCTCGTCAGGGTCCATCCACTCCAAGCTC |
| Pps-2 | TTCAGAGGTCTCTCTGACACTGGAATCGGCAGCAAAGG |
| Pgs-L | AGCGTGGGTCTCGCTCGACGCGTATCCATCCACTCCAAGCTC |
| *FTL8*-gRT1 | CTCTCAACGTCGGATTGCTTgttttagagctagaaat |
| *FTL8*-OsU3T1 | AAGCAATCCGACGTTGAGAGCggcagccaagccagca |
| Overexpression | |
| *SCL30*-CDS-Flag-F | catgccatggATGAGGAGGTACAGCCCACCAT |
| *SCL30*-CDS-Flag-R | actgggtcaccTCACTTGTCATCGTCATCCTTGTAGTCGATGTCATGAT  CTTTATAATCACCGTCATGGTCTTTGTAGTCGTCGCTGCGGGCAGG |
| *Promoter* | |
| *SCL30*-P-F | cgcggatccTCTATCCAGGCACAGTTGGATGTT |
| *SCL30*-P-R | cccaagcttTGTTTTGTCTGAAGCACATAACAGAA |
| Y-1-H | |
| *SCL30*-P-300-F | cccaagcttTGTAATCGAAAGCCCATAAGGC |
| *SCL30*-P-300-R | cgacgtcgacAGCCAATCATGCGACCCATC |
| *SCL30*-P-175-F | cccaagcttATCTTAGAGTGCCATCTAGTTCCT |
| *SCL30*-P-175-R | cgacgtcgacGACACAAACTCACTCTCTTTCTC |
| NBS-3×AAAAATCT-F | cccaagcttAAAAATCTAAAAATCTAAAAATCTgtcgacgtcg |
| NBS-3×AAAAATCT-R | cgacgtcgacAGATTTTTAGATTTTTAGATTTTTaagcttggg |
| pAbAi-seq-F | TTCAAGGAAACCGAAATCA |
| pAbAi-seq-R | TATGCTACAAAGGACCTAAT |
| AD-OsLHY-F | ccggaattcATGGAGATTAATTCCTCTGGTGAGG |
| AD-OsLHY-R | ccgctcgagTCATGTCGATGCTTCGCTCTC |
| DLuc | |
| pGreenII-SCL30pro-F | ccctttcgtcttcaagaattcATCTTAGAGTGCCATCTAGTTCCT |
| pGreenII-SCL30pro-R | ataatgccaggaatttctagaGACACAAACTCACTCTCTTTCTC |
| GST-LHY-F | ccgcgtggatccccggaattcATGGAGATTAATTCCTCTGGTGAGG |
| GST-LHY-R | ctcgagtcgacccgggaattcTCATGTCGATGCTTCGCTCTC |
| Y-2-H | |
| BD-OsSCL30-F | catgccatggATGAGGAGGTACAGCCCACCAT |
| BD-OsSCL30-R | ccggaattcTCAGTCGCTGCGGGCAGG |
| AD-DHT1-F | ccggaattcATGGCTCCGAAGAAGCGAAAGGCGG |
| AD-DHT1-R | cgcggatccAAATCCACCCTGAAAACCAGCA |
| Subcellular localization | |
| SCL30-GFP-F | GACGAGCTCGGTACCTCTAGAATGAGGAGGTACAGCCCACCAT |
| SCL30-GFP-R | TTCTCCTTTACTCATTCTAGAATCAGTCGCTGCGGGCAGG |
| LUC | |
| DHT1-LUC-F | AGATCGAATTCCATGGTCATGGCTCCGAAGAAGCGAAAG |
| DHT1-LUC-R | TTGGCGTCTTCCATGGCAAATCCACCCTGAAAACCAGCA |
| SCL30-nLUC-F | CGGGGGACGAGCTCGGTACCATGAGGAGGTACAGCCCACCATA |
| SCL30-nLUC-R | ACGAGATCTGGTCGACGTCGCTGCGGGCAGGG |
| BiFC | |
| SCL30-YN-F | TCCATCGATAGTACTGTCGACATGAGGAGGTACAGCCCACCAT |
| SCL30-YN-R | CTCCATCCCGGGAGCGGTACCGTCGCTGCGGGCAGG |
| DHT1-YC-F | GAGAACACGGGGGACTCTAGAATGGCTCCGAAGAAGCGAAAG |
| DHT1-YC-R | GTACATCCCGGGAGCGGTACCATCCACCCTGAAAACCAGCA |
| RT-PCR | |
| *Actin*-F | ACCACAGGTATTGTGTTGGACTC |
| *Actin*-R | AGAGCATATCCTTCATAGATGGG |
| *OsSCL30*-FP1 | GAGGTACAGCCCACCATATC |
| *OsSCL30*-RP1 | ATCAAAATGGAATCGACATGCC |
| *OsSCL30*-RP2 | CGCTGTAATAATCCTTTGGCAG |
| sRT-*OsFTL8*-A3SS-F | GATGCAGCGACGATGTCATT |
| sRT-*OsFTL8*-A3SS-R | TTGTACATCACCCGGAGCGT |
| qRT*-Hd3a*-F | GCTCACTATCATCATCCAGCATG |
| qRT*-Hd3a*-R | CCTTGCTCAGCTATTTAATTGCATAA |
| qRT*-RFT1*-F | TGACCTAGATTCAAAGTCTAATCCTT |
| qRT*-RFT1*-R | TGCCGGCCATGTCAAATTAATAAC |
| qRT*-Ehd1*-F | CCTACAGTGATTATGGCTTCA |
| qRT*-Ehd1*-R | GTGCTGCCAAATGTTGCTC |
| qRT*-OsLHY*-F | CCACCAGGGAAGTTCAAAAT |
| qRT*-OsLHY*-R | ATGGGACTTCTCGAAAGAGA |
| qRT*-OsRE1*-F | TGGAGAGCGACGAGGAGATA |
| qRT*-OsRE1*-R | TTCAGCCGGTTCTGCTCCT |
| qRT-*OsFTL8*-F | CGACGTGGTGGACCTGTTCT |
| qRT-*OsFTL8*-R | GGAGCATCTGGATCCACCAT |
| qRT-D14-F | GCTTCGGGACGGACCAGT |
| qRT-D14-F | TTGTCGTAGCGGCGGAAG |
| sRT*-OsLHY*-F | GAGGCAGAGCACAACAGGTT |
| sRT*-OsLHY*-R | CTGCACAGCTGTCTTTGTCC |
| sT*- RFT1*-F | TGACCTAGATTCAAAGTCTAATCCTT |
| sRT*-RFT1*-R | CTCCCTAAGGTTAGGGTTGCTT |
| sRT*- Hd3a* -F | GCGGCAATGACATGAGGACATT |
| sRT*- Hd3a*-R | CGCTGCAGTAGTACCAGGAATA |
| sRT*-OsRE1*-F | GCCCCCAACAACCTCAAGGAA |
| sRT*-OsRE1*-R | CATGTACGCCTTCTTCCGCT |
| sRT*-OsCCD7*-F | GGTCCGAATCGATCGAAGTGG |
| sRT*-OsCCD7*-R | GATCTTGTAGTGCGCCAGCAG |
| sRT*-OsLHY*-F1 | CCACCAGGGAAGTTCAAAAT |
| sRT*-OsLHY*-R1 | CGAAATACTACAAAGGTATGTAC |
| sRT*-OsLHY*-R2 | ATGGGACTTCTCGAAAGAGA |
| sT*- RFT1*-F1 | TGGCAATGACATGAGGACGTTC |
| sRT*-RFT1*-R1 | GCCACACAAAATTAACATGCATCC |
| sRT*-RFT1*-R2 | CTCCCTAAGGTTAGGGTTGCTT |
| sRT*- Hd3a* -F1 | GCGGCAATGACATGAGGACATT |
| sRT*- Hd3a*-R1 | GTATAACTTCTGGTCTCTGCACC |
| sRT*- Hd3a*-R2 | CGCTGCAGTAGTACCAGGAATA |
| sRT*-OsRE1*-F1 | CCACCCTCCAGAACGAGAACAA |
| sRT*-OsRE1*-R1 | GGAGAGTAGAGGAGAAATCGCC |
| sRT*-OsRE1*-R2 | GTGAAATGGTGCTTCTTCCCG |
| sRT*-CCD7*-F1 | GGTCCGAATCGATCGAAGTGG |
| sRT*-CCD7*-R1 | GGTCCGAATCGATCGAAGTGG |
| sRT*-CCD7*-R2 | GATCTTGTAGTGCGCCAGCAG |

## Table S2 Agronomic traits between Nip, gene editing lines *scl30-1* and *scl30-2*

| Traits | Nip | *scl30-1* | *scl30-2* |
| --- | --- | --- | --- |
| Head days (Days) | 65.30 ± 0.46 ^a^ | 59.80 ± 0.60 ^b^ | 60.00 ± 0.63 ^b^ |
| Height (cm) | 92.83 ± 1.37 ^a^ | 83.18 ± 1.11 ^c^ | 86.00 ± 1.33 ^b^ |
| Effective tiller number（No.） | 18.60 ± 2.65 ^b^ | 25.20 ± 1.99 ^a^ | 25.50 ± 2.69 ^a^ |
| Panicle length (cm) | 22.83 ± 0.45 ^a^ | 17.57 ± 0.47 ^c^ | 18.41 ± 0.56 ^b^ |
| Grain length (mm) | 7.56 ± 0.15 ^c^ | 7.96 ± 0.12 ^a^ | 7.80 ± 0.10 ^b^ |
| Grain width (mm) | 3.48 ± 0.07 ^a^ | 3.11 ± 0.08 ^b^ | 3.08 ± 0.10 ^b^ |
| Grain thickness (mm) | 2.37 ± 0.10 ^a^ | 2.19 ± 0.07 ^b^ | 2.16 ± 0.07 ^b^ |
| Grain length/grain width | 2.17 ± 0.04 ^b^ | 2.56 ± 0.08 ^a^ | 2.53 ± 0.08 ^a^ |
| 1000-grain weight (g) | 23.94 ± 0.62 ^a^ | 20.88 ± 0.46 ^b^ | 20.39 ± 0.55 ^b^ |
| Yield per plant (g) | 14.75 ± 0.58 ^a^ | 10.74 ± 0.50 ^b^ | 10.70 ± 0.45 ^b^ |

Note: Different letters after standard error indicate significant differences between treatments at *p* ≤ 0.05, as determined by one-way ANOVA analysis (n = 10).

## Table S3 Agronomic traits between Nip, *scl30-1* and overexpression lines

| Traits | Nip | *scl30-1* | OE#1 | OE#2 |
| --- | --- | --- | --- | --- |
| Head days (Days) | 63.70 ± 0.64 ^c^ | 57.60 ± 0.66 ^d^ | 67.80 ± 0.60 ^a^ | 67.30 ± 0.46 ^a^ |
| Height (cm) | 91.73 ± 2.26 ^c^ | 83.79 ± 1.42 ^d^ | 101.17 ± 2.08 ^a^ | 94.95 ± 2.00 ^b^ |
| Effective tiller number（No.） | 17.80 ± 1.66 ^a^ | 24.70 ± 1.85 ^b^ | 17.20 ± 1.721 ^a^ | 17.10 ± 1.87 ^a^ |
| Panicle length (cm) | 22.83 ± 0.45 ^a^ | 17.57 ± 0.47 ^d^ | 20.06 ± 0.51 ^b^ | 19.98 ± 0.47 ^b^ |
| Grain length (mm) | 7.46 ± 0.19 ^c^ | 7.85 ± 0.11 ^b^ | 8.13 ± 0.16 ^a^ | 8.12 ± 0.15 ^a^ |
| Grain width (mm) | 3.34 ± 0.12 ^a^ | 3.01 ± 0.10 ^b^ | 2.91 ± 0.05 ^b^ | 2.90 ± 0.06 ^b^ |
| Grain thickness (mm) | 2.31 ± 0.07 ^a^ | 2.11 ± 0.07 ^b^ | 2.01 ± 0.09 ^b^ | 2.06 ± 0.09 ^b^ |
| Grain length/grain width | 2.24 ± 0.07 ^c^ | 2.61 ± 0.11 ^b^ | 2.79 ± 0.07 ^a^ | 2.80 ± 0.08 ^a^ |
| 1000-grain weight (g) | 23.54 ± 0.67 a | 20.52 ± 0.39 c | 20.80 ± 0.48 b | 21.16 ± 0.46 b |
| Yield per plant (g) | 14.91 ± 0.59 ^a^ | 10.89 ± 0.54 ^c^ | 12.98 ± 0.53 ^b^ | 13.05 ± 0.47 ^b^ |

Note: Different letters after standard error indicate significant differences between treatments at *p* ≤ 0.05, as determined by one-way ANOVA analysis with 10 replicates.

## Table S4 Transcription factor prediction of *OsSCL30* promoter

| Type | Sequence | Location* | *Cis*-elements |
| --- | --- | --- | --- |
| Light-responsive elements | ATTAAT | 1409(+), 1436(+) | Box 4 |
|  | AATCTAATCC | 604(-) | ATCT-motif |
|  | TACGTG | 543(+) | G-box |
|  | TCTTAC | 254(-), 451(+) | TCT-motif |
|  | TCTCCCT | 980(+) | TCCC-motif |
|  | CAGCGTGTGGC | 2310(+) | GTGGC-motif |
|  | AGAAACTT | 517(+) | AE-box |
| Hormone-responsive elements | CGTCA | 241(-), 553(-), 1322(-) | CGTCA-motif |
|  | TGACG | 241(+), 553(+), 1322(-) | TGACG-motif |
|  | ACGTG | 542(+), 1324(-), 1691(-) | ABRE |
|  | AACGAC | 1376(-), 2333(+) | TGA-element |
|  | CCTTTTG | 1786(-) | P-box |
| Stress-responsive elements | ATTCTCTAAC | 455(+), 507(-) | TC-rich repeats |
|  | CAACTG | 2661(-) | MBS |
|  | CAACGG | 496(-), 2278(-) | CCAAT-box |
|  | AAACCA | 654(-), 1166(+), 1301(+) | ARE |
| Tissue-specific expression elements | GCCACT | 1366(-) | CAT-box |
|  | TGAGTCA | 1336(-) | GCN4-motif |

Note: *: The ATG is the start position. +, sense strand;-：antisense strand.

## Table S5 Transcription factor prediction of *OsSCL30* promoter

| Transcription factor family | *Position | Transcription factor | *Cis*-element |
| --- | --- | --- | --- |
| MYB | 1640(+) | *OsLHY* | AAAAATCT |
| GAGA | 846(-), 1558(+) | *OsGBP3* | GAGAGAGAGA |
| GAGA | 846(-), 1558(+) | *OsGBP1* | GAGAGAGAGA |
| Dof | 1392(-) | *OsDOF11* | AATGGAGAGAAAAAGTATTAA |
|  | 1178(-) |  | GAATAAAGGAAAAAGTATACT |
| ERF | 915(-), 912(-), 909(-), 906(-) | *FZP* | CCGCCG |
| AP2 | 915(-), 912(-), 909(-), 906(-) | *MFS1* | CCGCCG |
| AP2 | 915(-), 912(-), 909(-), 906(-) | *OsERF71* | CCGCCG |

Note: *: The ATG is the start position. +, sense strand;-：antisense strand.

# Supplementary figures

## Figure S1

Figure S1 The mutation sites for gene-editing and expression of overexpression lines of *OsSCL30*. A) Schematic diagram of *OsSCL30*. The blue arrows indicated the primers for splicing efficiency. The primer pairs of FP1 and RP1, and FP1 and RP2 were used for amplification of unspliced mRNA and spliced mRNA, respectively. The unfilled boxes, the filled boxes, lines, and red triangle represent UTR (5’-UTR and 3’-UTR), the CDS, introns, and editing sites, respectively. (+), and (-) represent the insertion sites and the deletion site at two *scl*30 gene-editing lines, which created by CRISPR/Cas9 system. respectively. Target 1 and Target 2 represent target site 1 and 2, respectively. B) CRISPR /Cas9 vector schematic. C) The sequences of Nipponbare and two gene-editing lines. D) Schematic diagram of *OsSCL30* gene overexpression vector. E) The expression level of *OsSCL30* in two overexpression lines. Data were expressed as the mean ± SD of three biological replicates.

## Figure S2

Figure S2 The day length and Effective accumulated temperature at the growing location in Changxing, Zhejiang. A) Day length at Changxing. Changes in day length at the growing location in Changxing, Zhejiang. Gray boxes represent the period above 13 h day length. Generally, field conditions in Changxing are supposed to be natural long-day (nLD) conditions. B) Effective accumulated temperature.

## Figure S3

Figure S3 Comparison of heading date and plant height among *scl30-1*, Nip and F_1_.

A-B) Heading date. C) Plant height. The histograms for heading date (D) and plant height (E). Nip: Nipponbare; *scl30-1*: gene editing line; F_1_: the cross generation of *scl30-1* and Nip; Scale bars = 10 cm. Data in D and E are presented as means ± SD of 10 samples). ** and ns indicated significantly difference and no difference, respectively (P < 0.05), as determined by Student’S t-test.

## Figure S4

Figure S4 *OsSCL30* promoter *cis*-elements interacted with OsLHY using Y1H. A) Test of bait yeast strain for AbA^r^ expression. B) The interaction of *cis*-element of *OsSCL30* and OsLHY.

## Figure S5

Figure S5 Classification of differentially expressed genes in KEGG enrichment analysis.

## Figure S6

Figure S6 Classification of differential splicing genes in KEGG enrichment analysis.

## Figure S7

Figure S7 The domains and structure of OsSCL30. A) The structure and domain of OsSCL30 from NCBI. B-C) The model alignment and structure of OsSCL30 from [SWISS-MODEL](https://swissmodel.expasy.org/repository) analysis.

## Figure S8

Figure S8 Schematic diagram of five target genes. The red arrows indicated the primers for splicing efficiency. The primer pairs of FP1 and RP1, and FP1 and RP2 were used for amplification of unspliced mRNA and spliced mRNA, respectively. The gray boxes, blue boxes, lines, represent UTR (5’-UTR and 3’-UTR), the CDS, introns, and editing sites, respectively.

## Figure S9

Figure S9 Splicing effect of target genes between the WT and *scl30-1* mutant. A, B and C represent relative transcript of spliced mRNA, relative transcript of unspliced mRNA, and spliced mRNA/total mRNA of target gene, respectively. Data are presented as means ± SD of three biological replicates. Significant differences were identified at 5％(*) and 1% (**) probability levels by Student’S t-test．

## Figure S10

Figure S10 Multiple alignment of TFL-like proteins in rice. Amino acid residues conserved in all proteins are shaded in black.

## Figure S11

Figure S11 Haplotype network of *OsSCL30*. Circle size is proportional to the number of samples for a given haplotype.

## Figure S12

Figure S12 The schematic model for OsSCL30 modulated on heading date and plant height in rice.
